# Deciphering the O-Glycosylation of HKU1 Spike Protein With the Dual-Functional Hydrophilic Interaction Chromatography Materials

**DOI:** 10.3389/fchem.2021.707235

**Published:** 2021-08-13

**Authors:** Yun Cui, Xuefang Dong, Xiaofei Zhang, Cheng Chen, Dongmei Fu, Xiuling Li, Xinmiao Liang

**Affiliations:** ^1^ School of Biological Engineering, Dalian Polytechnic University, Dalian, China; ^2^ Key Lab of Separation Science for Analytical Chemistry, Dalian Institute of Chemical Physics, Chinese Academy of Sciences, Dalian, China

**Keywords:** HKU1, spike glycoprotein, enrichment, O-glycosylation sites, O-glycosylation abundance

## Abstract

HKU1 is a human beta coronavirus and infects host cells *via* highly glycosylated spike protein (S). The N-glycosylation of HKU1 S has been reported. However, little is known about its O-glycosylation, which hinders the in-depth understanding of its biological functions. Herein, a comprehensive study of O-glycosylation of HKU1 S was carried out based on dual-functional histidine-bonded silica (HBS) materials. The enrichment method for O-glycopeptides with HBS was developed and validated using standard proteins. The application of the developed method to the HKU1 S1 subunit resulted in 46 novel O-glycosylation sites, among which 55.6% were predicted to be exposed on the outer protein surface. Moreover, the O-linked glycans and their abundance on each HKU1 S1 site were analyzed. The obtained O-glycosylation dataset will provide valuable insights into the structure of HKU1 S.

## Introduction

The human HKU1 coronavirus (CoV) was first discovered in Hong Kong in 2004 and found to cause prevalent respiratory diseases (Woo et al., 2005). HKU1 is a kind of beta coronavirus (β-CoV), which includes the severe acute respiratory syndrome (SARS-CoV), Middle East respiratory syndrome (MERS-CoV), and SARS-CoV-2 (Christian et al., 2004; Woo et al., 2009; Zaki et al., 2012; Hoffmann et al., 2020). The CoV spike (S) protein is a large type I transmembrane glycoprotein, and it mediates virus entry to the host cells (Heald-Sargent and Gallagher, 2012). The S protein has two subunits: the S1 subunit is responsible for receptor binding, whereas the S2 subunit facilitates membrane fusion (Millet and Whittaker, 2015; Kirchdoerfer et al., 2016). Specifically, S1 contains two independent domains: an amino (N)-terminal domain (NTD) and a carboxy (C)-terminal domain (CTD) (Peng et al., 2011). Several β-CoVs, including mouse hepatitis virus, human CoV OC43, and bovine CoV (BCoV), use their NTDs to bind receptor protein (Peng et al., 2011; Peng et al., 2012). By contrast, HKU1 uses its CTD to bind to receptors (Qian et al., 2015), similar to SARS-CoV, MERS-CoV, and SARS-CoV-2 (Li et al., 2003; Mou et al., 2013; Hoffmann et al., 2020). Glycosylation contributes significantly to the conformation of the S protein and therefore profoundly affects receptor binding (Fung and Liu, 2018). The S protein of HKU1 is highly N-glycosylated, and 29 N-glycosylation sites have been deciphered (Watanabe et al., 2020). The glycan shield density of the HKU1 S protein is considerably higher than that of SARS-CoV and MERS-CoV (Watanabe et al., 2020).

Except for N-glycosylation, viral O-glycosylation plays pivotal roles in viral entry, propagation, and immune recognition (Bagdonaite et al., 2015; Iversen et al., 2016; Olofsson et al., 2016; Stone et al., 2016). The O-glycosylation of viral surface proteins on human cytomegalovirus, Epstein–Barr virus (Bagdonaite et al., 2016), and hepatitis C virus (Brautigam et al., 2012) have been extensively reported. More importantly, the O-linked glycans on human immunodeficiency virus type 1 can shield against one category of broadly neutralizing antibodies (Silver et al., 2020). In addition, the O-glycosylation on viral glycoproteins can be developed as the epitopes for the potential development of subunit vaccines (Olofsson et al., 2016). However, the O-glycosylation of HKU1 is scarcely reported, and the related virology research on O-glycosylation of S protein is severely hindered.

The identification of O-glycosylation is more challenging than that of N-glycosylation, owing to the lack of conserved O-glycosylation site sequon and consistent O-linked glycan cores, inefficient O-linked glycan-specific glycosidases, and extremely low O-glycosylation stoichiometry (You et al., 2018). Thus far, hydrazide chemistry (Yang et al., 2017), “SimpleCell” method (Steentoft et al., 2011), and lectin affinity chromatography methods (Anan et al., 2019) have been adopted to enrich O-glycopeptides. However, hydrazide chemistry method always destroys the intact glycan structure during the oxidation step. The “SimpleCell” technology blocks the natural elongation of O-linked glycans but eliminates their heterogeneity (Steentoft et al., 2011). The lectin affinity chromatography method can only enrich individual O-linked glycan and lacks universality (Narimatsu et al., 2019; Singh et al., 2020).

Hydrophilic interaction liquid chromatography (HILIC) has been widely adopted to enrich N-glycopeptides with no bias to glycan structures (Cao et al., 2014; Hoffmann et al., 2016; Shao et al., 2016; You et al., 2018; Qing et al., 2020). In our previous work (Dong et al., 2017), dual-functional histidine-bonded silica (HBS) HILIC materials were prepared, and they demonstrated the selective enrichment of N-glycopeptides from human serum (Dong et al., 2017; Qin et al., 2019). Thus, it was expected that HBS materials can be applied for the enrichment of O-glycopeptides from HKU1 S. To achieve this goal, we first developed the enrichment method of O-glycopeptides based on HBS by optimizing different enrichment conditions with bovine fetuin as the model glycoprotein. This newly developed method was further validated by enriching O-glycopeptides from a mixture of bovine fetuin and albumin bovine serum digests, and commercial ZIC-HILIC materials were used for comparison. Finally, O-glycosylation of HKU1 S1 was comprehensively characterized including the O-glycosylation site identification, glycosylation site distribution, exposure ratio prediction, and O-linked glycan analysis. We believe that deciphering O-glycosylation will provide a significant complement to glycosylation for HKU1 S.

## Materials and Methods

### Reagents and Materials

Bovine fetuin, albumin bovine serum (BSA), trypsin, elastase, and chemical reagents of iodoacetamide (IAA), 1,4-dithiothreitol (DTT), acetic acid (HAc), ammonium bicarbonate (NH_4_HCO_3_), ammonia water (NH_3_·H_2_O), urea, and the zwitterionic hydrophilic interaction liquid chromatography (ZIC-HILIC) materials were obtained from Sigma (St. Louis, MO). HKU S1 (expressed from HEK293 cells, the purity ＞ 93.2%) was purchased from Sino Biological. PNGase F was purchased from New England Biolabs (Ipswich, MA). Acetonitrile (ACN, HPLC grade) was from Merck (Darmstadt, Germany). Formic acid (FA) was obtained from Honeywell (Shanghai, China). Trifluoroacetic acid (TFA) was obtained from Macklin (Shanghai, China). Pure water used in all experiments was purified with a Milli-Q system (Millipore, Milford, MA). GELoader tips were purchased from Eppendorf (Hamburg, Germany). C18 AQ materials were obtained from Acchrom (Beijing, China). Histidine-bonded silica (HBS) materials were homemade.

### Digestion of Proteins

Each protein (BSA, bovine fetuin, and HKU1 S1) of 1 mg was denatured with 100 μl 6 M urea in 50 mM NH_4_HCO_3_ for 3 h, and then 20 μl DTT (200 mM) was added for reduction at 56°C for 45 min. After adding 40 μl IAA (200 mM) in dark for 30 min, fivefold volume of 50 mM NH_4_HCO_3_ was added to the solution and then mixed with different enzymes. Trypsin was added for BSA digestion at an enzyme/protein mass ratio of 1:25 (w/w). Bovine fetuin was first digested by elastase at an enzyme/protein ratio of 1:40 (w/w), and then PNGase F was added at 37°C overnight to remove N-glycans. HKU1 S1 was first digested by trypsin and chymotrypsin with the enzyme/protein ratio of 1:20 (w/w), and then PNGase F was used to remove N-glycans at 37°C overnight. Finally, the protein digests were collected and lyophilized to dryness.

### Enrichment of O-Glycopeptides From Protein Digests

#### Optimization of HBS-Based Enrichment Conditions for O-Glycopeptides

Although the HBS-based enrichment method for N-glycopepitdes has been established, the strategy for O-glycopeptide enrichment has not been developed. Thus, we investigated the effect of different ACN contents (from 50 to 80%, v/v), pH value (adjusting by FA or NH_4_HCO_3_), and types of acid additive (FA, HAc, and TFA) on the O-glycopeptide enrichment efficiency. Bovine fetuin was selected as model protein, and the typical O-glycopeptides with m/z 1300.2193 (3+) and 1440.0979 (2+), and non-glycosylated peptides with m/z 1122.5524 (1+) and 1213.5851 (1+) were selected to evaluate the enrichment performance. The detailed information of the peptide sequence and the glycan structure of the typical O-glycopeptides is shown in Supplementary Table S1, signed with green.

#### Enrichment of O-Glycopeptides From Bovine Fetuin Digests With Optimized Method

One milligram of HBS materials suspended in 20 μl of ACN was packed into a GELoader tip. The tip was activated with 50% ACN/0.1% TFA (30 μl × 3) and equilibrated with 80% ACN/0.1% TFA (30 μl × 3) successively. Then, 10 μg bovine fetuin digests in 80% ACN/0.1% TFA was loaded on the HBS materials and then washed with 20 μl of 80% ACN/0.1% TFA twice. Subsequently, the materials were eluted with 20 μl of 10% NH_3_·H_2_O, then the eluent was collected and dried to remove NH_3_·H_2_O, followed by redissolving in 20 μl of 50% ACN/0.1% FA for ESI Q-TOF MS analysis.

#### Enrichment of O-Glycopeptides From Mixed Standard Protein Digests

The digest mixture of fetuin and BSA at a mass ratio of 1:20, 1:200 (10 μg bovine fetuin) was mixed with 1 mg HBS in 500 μl and 2 mg HBS in 4 ml of 80% ACN/0.1% TFA, respectively. The obtained solution was shaken for 40 min, followed by centrifugation at 10,000 g for 2 min. Then, the supernatant was removed, and the precipitation was washed with 80% ACN/0.1% TFA (250 μl × 3 for ratio of 1:20, and 1 ml × 3 for ratio of 1:200, respectively) to remove the non-glycosylated peptides. Subsequently, the precipitation was transferred into a GELoader tip, respectively, and eluted with 30 μl of 10% NH_3_·H_2_O. The eluent was collected and dried to remove NH_3_·H_2_O, followed by redissolving in 30 μl of 50% ACN/0.1% FA for analysis by ESI Q-TOF MS.

In comparison, the enrichment with ZIC-HILIC was carried out as previously described (Huang et al., 2020), with relevant modification. The digest mixture of fetuin and BSA at a ratio of 1:20 (w/w) was mixed with 1 mg ZIC-HILIC in 500 μl of 80% ACN/0.2% TFA. The obtained solution was shaken for 10 min, followed by centrifugation at 10,000 g for 2 min. After removing the supernatant, the precipitation was washed with 80% ACN/0.2% TFA (250 μl × 3) to remove the non-glycosylated peptides. Then, the precipitation was transferred into a GELoader tip and eluted with 20 μl of 30% ACN/2% FA to obtain the O-glycopeptides.

#### Enrichment of O-Glycopeptides From HKU1 S1

Five milligrams of HBS was suspended in ACN and packed into a GELoader tip. The tip was activated with 50% ACN/0.1% TFA (30 μl × 3) and equilibrated with 80% ACN/0.1% TFA (30 μl × 3) successively. Then, 50 μg HKU1 S1 digests in 100 μl of 80% ACN/0.1% TFA was loaded on the HBS. The materials were washed with 80% ACN/0.1% TFA (30 μl × 3), and subsequently eluted with 40 μl of 10% NH_3_·H_2_O. The eluent was collected and dried for further liquid chromatography-tandem mass spectrometry (LC-MS/MS) analysis.

### Mass Spectrometry Analysis

The enriched O-glycopeptides from bovine fetuin digests were analyzed on a nano-ESI-Q-TOF mass spectrometer (Waters, Manchester, United Kingdom) with collision-induced dissociation (CID) in a positive mode. Full scan MS data were obtained at m/z 600–1700.

The enriched bovine fetuin and HKU1 S1 O-glycopeptides were separated and characterized using Q-Exactive Orbitrap coupled with Accela 600 HPLC system (Thermo, CA, United States), respectively. For the separation of peptides with reverse-phase liquid chromatography, 0.1% FA (pH 2.59) aqueous solution and ACN/0.1% FA were used as mobile phases A and B, respectively. The analytical column with an inner diameter of 75 μm was packed in-house with C18 AQ particles (3 μm, 120 Å) to 12 cm length. The flow rate was set at 600 nl/min. Gradient elution was performed with 2–8% B in 0.2 min, 8–50% B in 45 min, 50–90% B in 0.5 min, and 90% B in 5 min. Full mass scans were carried out on the Orbitrap with acquisition range from m/z 500 to 1500 (R = 70,000 at m/z 400). The 20 most intense ions from the full scan were selected for fragmentation *via* higher-energy collisional dissociation (HCD) in the ion trap. The dynamic exclusion function was set as follows: repeat count 1, repeat duration 30 s, and exclusion duration of 60 s.

### Data Analysis

All the RAW data files obtained from Orbitrap were searched against the database, using Byonic software (version 3.6.0, Protein Metrics, Inc.). The mass tolerance for precursors and fragment ions was set at 10 and 20 ppm, respectively. The O-glycans database was composed with 15 common O-glycans according to Zhao et al. (2020): [HexNAc(1)Hex(1), HexNAc(1)Hex(1)Fuc(1), HexNAc(2)Hex(1), HexNAc(1)Hex(1)NeuAc(1), HexNAc(2)Hex(2), HexNAc(2)Hex(1)NeuAc(1), HexNAc(1)Hex(1)NeuAc(2), HexNAc(2)Hex(2)NeuAc(1), HexNAc(3)Hex(1)NeuAc(1), HexNAc(2)Hex(2)Fuc(1)NeuAc(1), HexNAc(2)Hex(2)NeuAc(2), HexNAc(2)Hex(1)Fuc(1), HexNAc(1)Hex(2), HexNAc(1) NeuAc(1), and HexNAc(1)Hex(2)NeuAc(1)]. The fixed modification was carbamidomethyl (C), and variable modifications included oxidation (M), acetyl (protein N-term), and deamidation (N). Trypsin and chymotrypsin were set as the specific proteolytic enzymes with up to two missed cleavages allowed. Peptides with charge states of 2, 3, and 4 were chosen for further fragmentation. The FDR were all set as <1%. Moreover, the data were searched against reverse and contaminant sequences.

## Results and Discussion

### Optimization of HBS-Based Enrichment Conditions for O-Glycopeptides

**FIGURE 1:**
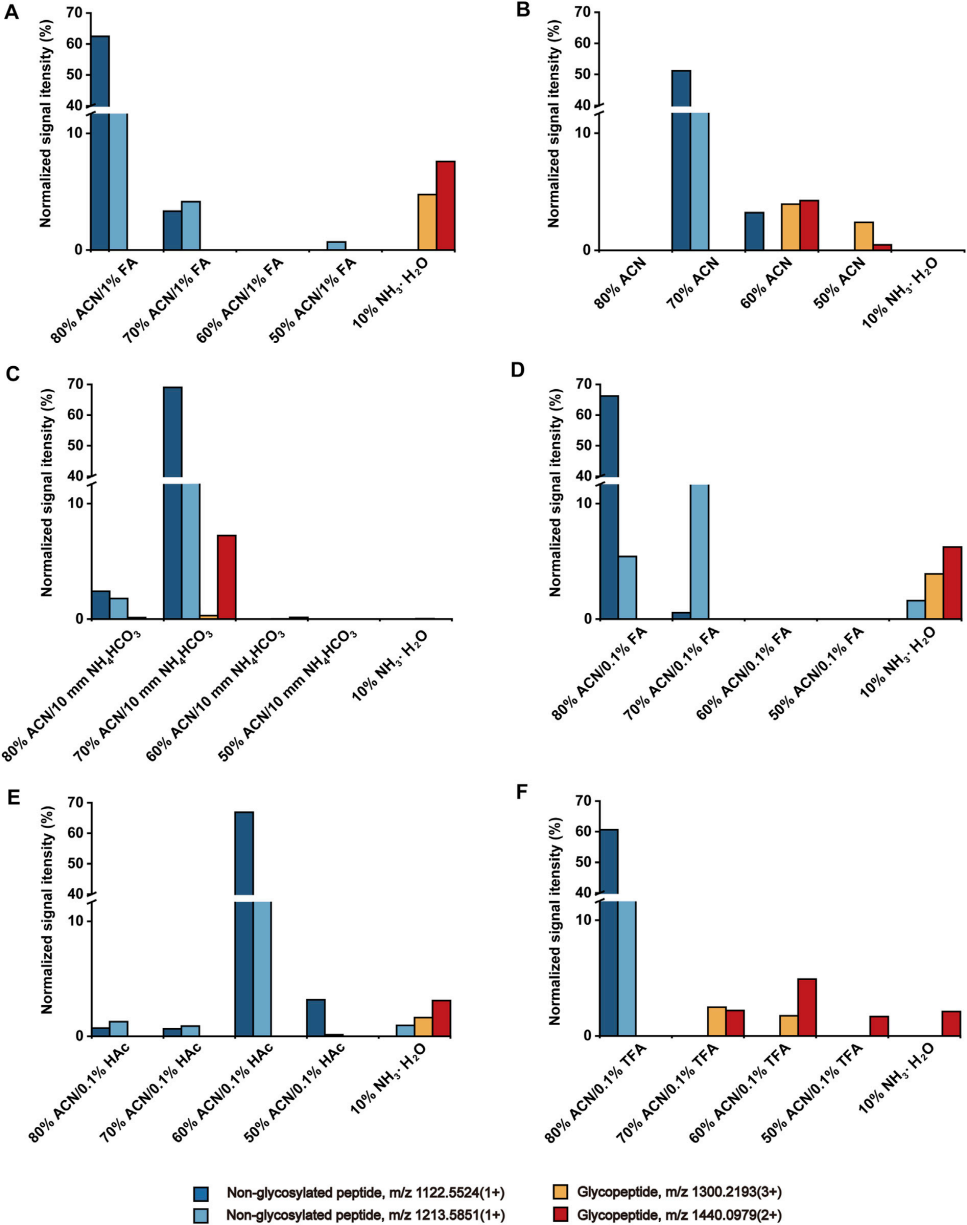
Effect of ACN content **(A)**, solution pH **(B, C)**, and types of acid additive **(D–F)** to the retention of peptides on HBS materials.

In the HILIC mode, the content of organic concentration determines the elution strength of the solvent, which affects solute retention on the stationary phase (Buszewski and Noga, 2012). Herein, the effect of different ACN contents (50–80%) on the O-glycopeptide enrichment on HBS was investigated under the same pH condition (containing 1% FA). As shown in Figure 1A, the non-glycosylated peptides were reserved from 50 to 80% ACN fractions, and O-glycopeptides were reserved in the elution fraction only. The result demonstrated that the retention of peptides on HBS possesses notable characteristics of HILIC, and O-glycopeptides can be retained strongly on HBS.

HBS with dual-functional characteristics displays hydrophilicity and switchable surface charge at different pH (Dong et al., 2017). To investigate the effect of pH on the enrichment of O-glycopeptides, we evaluated acidic, neutral, and basic ACN/H_2_O solutions. Compared with the acidic condition (Figure 1A), the co-elution of O-glycopeptides and non-glycosylated peptides was observed in neutral 60% ACN fraction (Figure 1B). When the solution was adjusted to a basic condition with 10 mM NH_4_HCO_3_ (Figure 1C), the O-glycopeptide with m/z 1300.2193 (3+) was almost undetectable even after the elution of 10% NH_3_·H_2_O. We collected the loading effluent and used the HBS materials to enrich the O-glycopeptides again. The O-glycopeptides with m/z 1300.2193 (3+) and 1440.0979 (2+) can be observed after the enrichment (Supplementary Figure S1). This finding demonstrated that the basic condition is unsuitable for O-glycopeptide enrichment with HBS materials because the positively charged O-glycopeptides are captured by the hydrophilic interaction of HBS materials under acidic condition, whereas HBS materials and O-glycopeptides are both negatively charged under basic condition, and O-glycopeptides cannot be captured on HBS. Therefore, an acidic solution is optimal for the O-glycopeptide enrichment on HBS.

Given that the acidic solution is favorable to the enrichment of O-glycopeptides on HBS, three types of acid additives (FA, HAc, and TFA) with 0.1% v/v to the solution were evaluated and compared. As shown in Figures 1D,E, non-glycosylated peptide m/z 1213.5851 (1+) was co-enriched with O-glycopeptides in the eluted solution, indicating that neither FA nor HAc is an optimal additive for O-glycopeptide enrichment. When the solution was added with TFA (Figure 1F), the non-glycosylated peptides flowed out to the 80% ACN fraction completely. In addition, O-glycopeptides occurred in the following fractions without any co-enrichment of non-glycosylated peptides. Although 1% FA as an acid additive facilitated the enrichment of O-glycopeptides significantly (Figure 1A), the efficiency of the removal of non-glycosylated peptides was greater with 0.1% TFA addition. Consequently, 0.1% TFA was selected as the acid additive for the following study.

### Enrichment of O-Glycopeptides From Bovine Fetuin Digests

**FIGURE 2:**
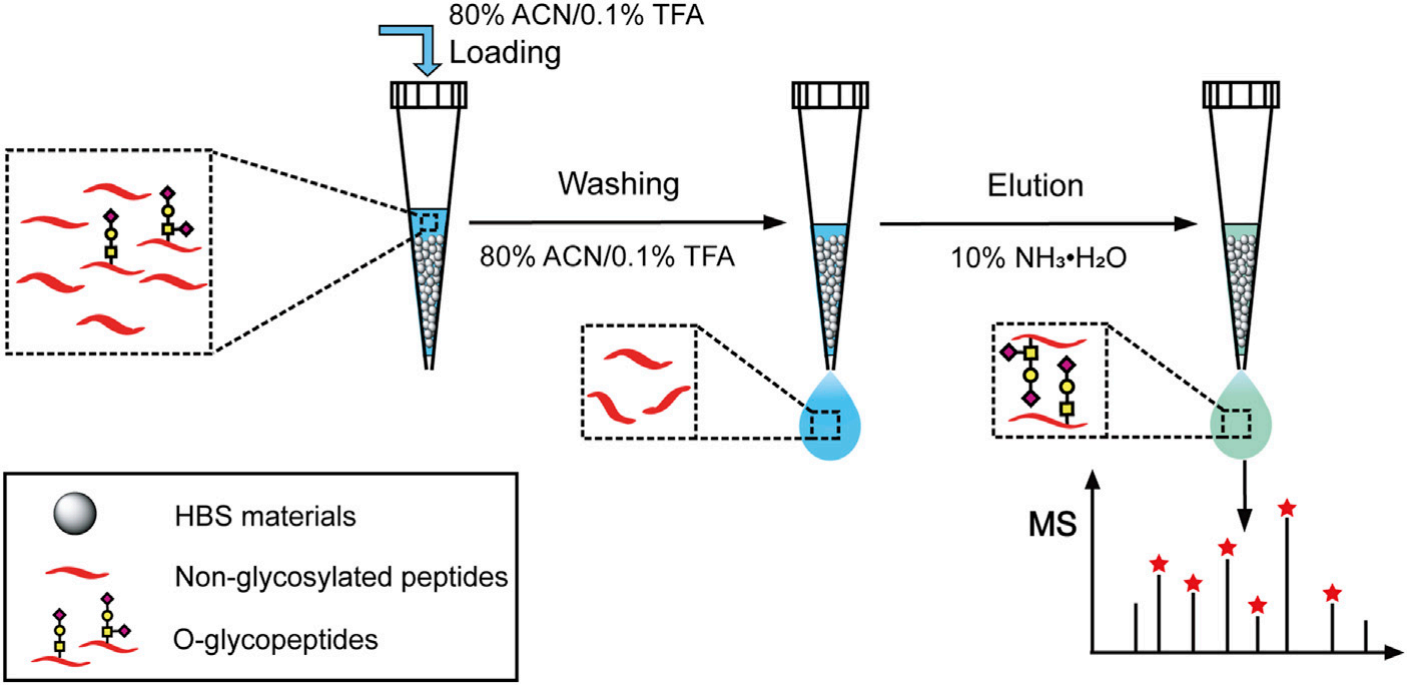
Schematic illustration for the O-glycopeptide enrichment with the dual-functional histidine-bonded silica materials.

**FIGURE 3:**
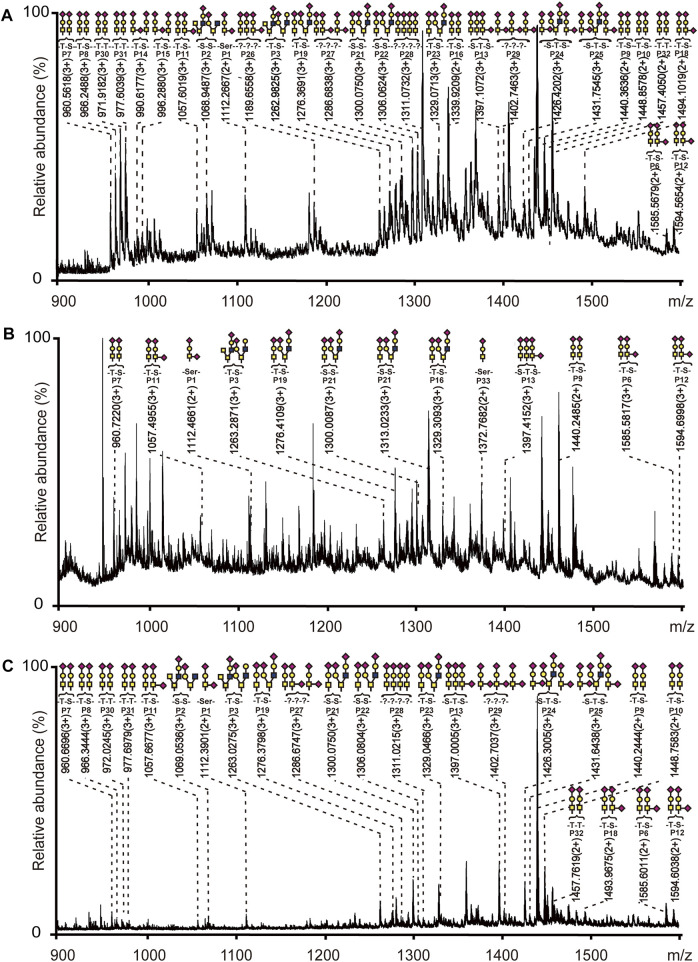
Mass spectra of digest mixture after enrichment with HBS or ZIC-HILIC materials. **(A)** Bovine fetuin and BSA at a mass ratio of 1:20 enriched with HBS. **(B)** Bovine fetuin and BSA at a mass ratio of 1:20 enriched with ZIC-HILIC. **(C)** Bovine fetuin and BSA at a mass ratio of 1:200 enriched with HBS. Glycopeptides are marked with their glycan structures: 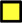, GalNAc; 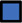, GlcNAc; 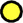, galactose; and 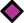, N-acetylneuraminic acid (Neu5Ac). The detailed information about the peptide sequences and glycosylation is shown in Supplementary Table S1.

Bovine fetuin, a glycoprotein containing sialylated N-linked and O-linked glycans, was used to evaluate the specificity and selectivity of HBS materials for O-glycopeptide enrichment. Based on the above optimized conditions, a process for O-glycopeptide enrichment was developed (Figure 2). The bovine fetuin digests in 80% ACN/0.1% TFA were loaded onto HBS, washed twice with 80% ACN/0.1% TFA to remove the non-glycosylated peptides, and then eluted with 10% NH_3_·H_2_O. With this optimized method, 32 O-glycopeptides were identified from the bovine fetuin digests (Supplementary Figure S2A). Supplementary Table S1 shows the details of these enriched O-glycopeptides. Further investigation was carried out with the digest mixture of bovine fetuin and BSA at different mass ratios to evaluate the enrichment selectivity of HBS to O-glycopeptides. No O-glycopeptide signal was observed from the desalted digest mixture at a ratio of 1:20 (w/w) without any enrichment (Supplementary Figure S2B). By comparison, 28 O-glycopeptides were identified after HBS enrichment from the same ratio of the digest mixture (Figure 3A). Commercial ZIC-HILIC was also used for the enrichment of O-glycopeptides, and 13 O-glycopeptides were detected from the same ratio of 1:20 (w/w) after enrichment (Figure 3B). The high ratio of the digest mixture was further investigated for the enrichment on HBS, and at the mass ratio of 1:200 (w/w), the HBS materials still showed a high selectivity, with 24 O-glycopeptides identified (Figure 3C). These results demonstrated that HBS materials have outstanding anti-interferential abilities, good selectivity, and specificity to O-glycopeptides.

### Validation of the Enrichment Method

In addition to selectivity, the reproducibility, recovery, limit of detection (LOD), and adsorption capacity are important parameters required to assess the developed enrichment method. The reproducibility of the optimized method was evaluated with bovine fetuin. The number of enriched O-glycopeptides was 31, 32, and 32 for three replicates. The recovery was measured by using the stable-isotope dimethyl labeling method (Boersema et al., 2009), and the recovery of typical two O-glycopeptides from bovine fetuin was over 93.9% (Supplementary Table S2), higher than that of ZIC-HLIC 84.3% (Supplementary Table S3). Given the lack of a standard O-glycopeptide, a standard sialylated glycopeptide (m/z 1433.2025) was used to test the LOD (S/N = 3), which reached 6.88 fmol/μl (Supplementary Figure S3). In addition, the adsorption capacity of HBS for bovine fetuin was 201.6 mg/g (Supplementary Figure S4). Thus, the optimized method in this study can be applied for the O-glycopeptide enrichment in complex samples.

### Comprehensive O-Glycosylation Analysis of HKU1 S Protein

#### Novel Strategy for Deciphering the O-Glycosylation of HKU1 S

**FIGURE 4:**
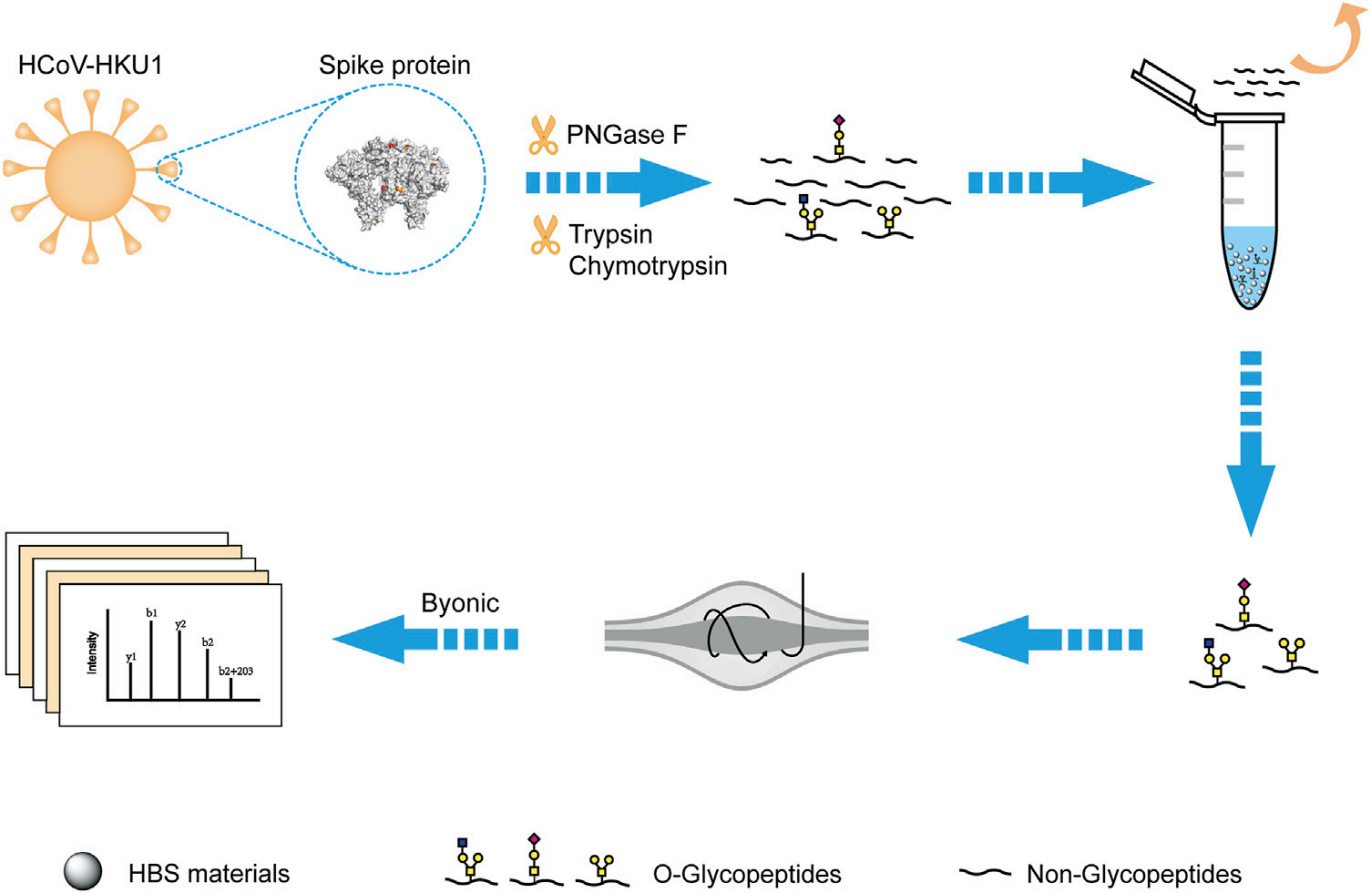
Schematic workflow for deciphering the O-glycosylation of HKU1 S1 with the dual-functional hydrophilic interaction chromatography materials. The PDB code is 5I08 for the structure of HKU1 S1.

Inspired by the O-glycopeptide enrichment efficiency on HBS, we developed a novel strategy for deciphering the O-glycosylation of HKU1 S. As shown in Figure 4, the recombinant HKU1 S1 was digested by trypsin, chymotrypsin, and PNGase F successively for digestion into a peptide sample and removal of N-linked glycans. The O-glycopeptides can be captured by the dual-functional HBS materials in the acidic condition and then released in the basic condition. The enriched O-glycopeptides were analyzed by LC-MS/MS. Searching the acquired data against the Byonic provided the identification information for further analysis. The efficiency of PNGase F to remove N-glycans was validated with bovine fetuin digests, and the result is shown in Supplementary Figure S5. The N-glycopeptides were successfully removed after PNGase F digestion. The detailed information of the typical N-glycopeptides and de-Nglycan peptides of bovine fetuin is shown in Supplementary Table S4. HCD and electron transfer dissociation are supplementary fragmentation types of O-glycosylation characterization in MS/MS (Yang et al., 2019; Riley et al., 2020). However, given the instrument limitation, we only used HCD to fragment the O-linked glycopeptides in this study. The stepped collision energy for HCD 20–30%, was set for sufficient fragmentation, and the MS/MS spectra were validated manually.

#### O-Glycosylation Site Identification and Distribution on HKU1 S

**FIGURE 5:**
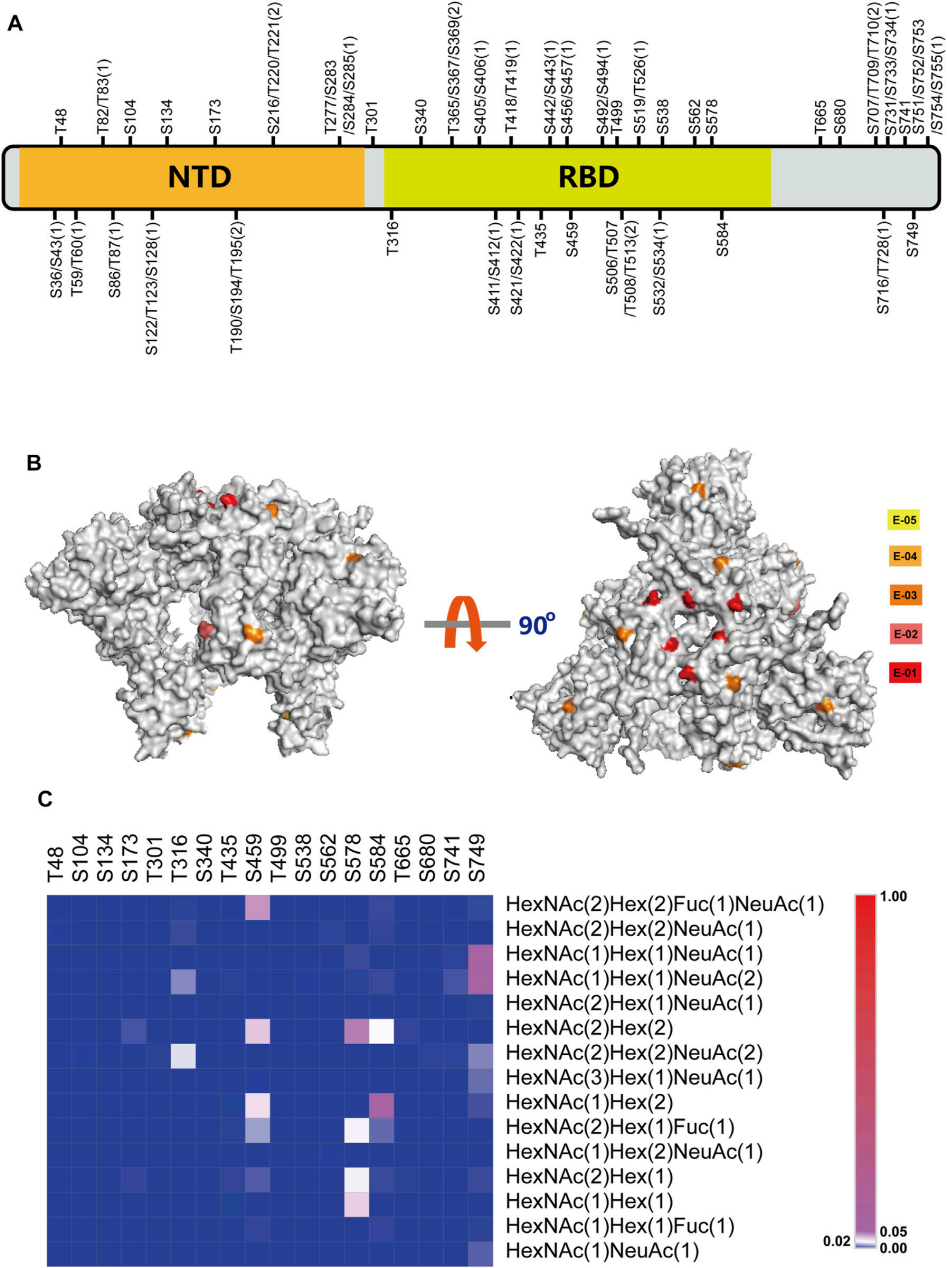
Comprehensive O-glycosylation analysis of HKU1 S1 protein. **(A)** Schematic representation of O-glycosylation sites (OGSs) identified on HKU1 S1. The ambiguous identified OGSs are labeled with potential S/T with the possible number of OGSs in bracket. N-terminal domain (NTD) is labeled in orange, and the receptor-binding domain (RBD) is labeled in lime green. Detailed information about the peptide sequences and glycosylation is shown in Supplementary Table S5. **(B)** The normalized O-glycosylation abundance on unambiguous OGSs mapped on HKU1 S1 (PBD code: 5I08). The normalized O-glycosylation abundance was calculated by dividing the O-glycosylation abundance on each O-glycosylation site by the total O-glycosylation abundance of HKU1 S1. Five orders of magnitudes were selected to label the normalized sialylated O-glycosylation abundance with yellow (E-05), bright orange (E-04), orange (E-03), deep salmon (E-02), and red (E-01), respectively. **(C)** The distribution of site-specific O-linked glycans on the individual O-glycosylation site.

Based on the developed novel strategy, the study for the O-glycosylation of HKU1 S1 was carried out, and 46 O-glycosylation sites were identified (Figure 5A). Among the identified O-glycosylation sites, 18 were unambiguously identified. Supplementary Figure S6 shows the corresponding LC-MS/MS b and y product ion fragments. All the O-glycosylation sites of HKU1 S1 were reported for the first time in this study. Compared with the 25 reported total O-glycosylation sites on SARS-CoV-2 S protein (Bagdonaite et al., 2021), the number of O-glycosylation sites on HKU1 S1 was higher. Furthermore, the distribution of O-glycosylation sites on two functional domains, namely, NTD and CTD, was investigated. CTD was reported as the receptor binding domain (RBD) of HKU1. A total of 14 and 22 O-glycosylation sites were distributed on NTD and RBD, respectively. These results showed that the O-glycosylation sites were not evenly but region-specifically distributed on HKU1 S1.

#### O-Glycosylation Analysis of HKU1 S1

The O-glycosylation site ratio of HKU1 S1, exposure degree of O-glycosylation on the HKU1 S1 outer surface, and exposure ratio on RBD were investigated. We calculated the O-glycosylation site ratio of HKU1 S1 by dividing the total number of amino acids by the number of O-glycosylation sites. The calculated O-glycosylation site ratio was 6.2% (46/747), whereas the N-glycosylation site ratio was 3.8% (29/747). Based on the protein surface accessibility and secondary structure predictions (http://www.cbs.dtu.dk/services/NetSurfP-1.1/), 20 O-glycosylation sites were predicted to be exposed on the HKU1 S1 outer surface (Supplementary Table S6), among which 10 were unambiguously exposed. The exposure degree of O-glycosylation sites on HKU1 S1 was 55.6% (10/18), which was slightly higher than that of SARS-CoV-2 S (52.4%, Bagdonaite et al., 2021). Particularly, the exposure ratio of O-glycosylation sites on RBD of HKU1 was 60% (6/10).

#### Mapping of Relative Normalized Abundances of O-Glycosylation on the 3D Model of HKU1 S1

To further explore the specific-site O-glycosylation on different sites, we investigated the relative normalized abundance of O-glycosylation on HKU1 S1 with visual models (PDB ID: 5I08). The normalized O-glycosylation abundance was calculated by dividing the O-glycosylation abundance on each O-glycosylation site by the total O-glycosylation abundance of HKU1 S1. We defined five magnitudes to represent different normalized O-glycosylation abundances as denoted in the keys (Figure 5B). As shown in Figure 5B, the RBD on the “head” of the subunit exhibited abundant O-glycosylation, especially the binding domain with receptor, but less exposed O-glycosylation on NTD.

#### Heat Map of the Total Relative Normalized Abundance of O-Glycosylation on HKU1 S1

In addition to the identification of O-glycosylation sites on the HKU1 S1 protein, the O-linked glycans were recognized. After searching the database with the 15 most common O-linked glycans, the distribution and normalized abundance of site-specific O-linked glycans of HKU1 S1 were mapped (Figure 5C). HexNAc(2)Hex(2), HexNAc(1)Hex(2), HexNAc(1)Hex(1)NeuAc(1), and HexNAc(1)Hex(1)NeuAc(2) were the top four most abundant O-linked glycans. Furthermore, the RBD displayed more O-glycosylation abundance on certain sites, such as S459, S578, and S584. K80 is the key residue for the HKU1 S protein to bind to 9-O-acetylated sialic acids from host cells (Hulswit et al., 2019). We identified that T82 or T83, which is adjacent to K80, is O-glycosylated. This may suggest hints between O-glycosylation and receptor binding. Overall, abundant O-glycosylation occurs on HKU1 S1, which also exhibits the micro- and macro-heterogeneity of O-glycosylation.

## Conclusion

In summary, a comprehensive study of O-glycosylation of the HKU1 S protein S1 subunit was carried out, and 46 O-glycosylation sites were identified, among which 18 were unambiguously identified. All of the O-glycosylation sites were reported for the first time in this study. The novel O-glycosylation information will give insights to the microstructure of the HKU1 S protein, thus facilitating the development of a potential HKU1 vaccine.

## Data Availability

The datasets presented in this study can be found in online repositories. The names of the repository/repositories and accession number(s) can be found below: ProteomeXchange, PXD025967.
