# Supplementary material for: Deciphering the O-Glycosylation of HKU1 Spike Protein With the Dual-Functional Hydrophilic Interaction Chromatography Materials
Source: Front Chem. 2021 Aug 13;9:707235. doi: 10.3389/fchem.2021.707235 (PMC8414140; doi:10.3389/fchem.2021.707235)
Supplement: Supplementary file 1 [file DataSheet1.pdf]

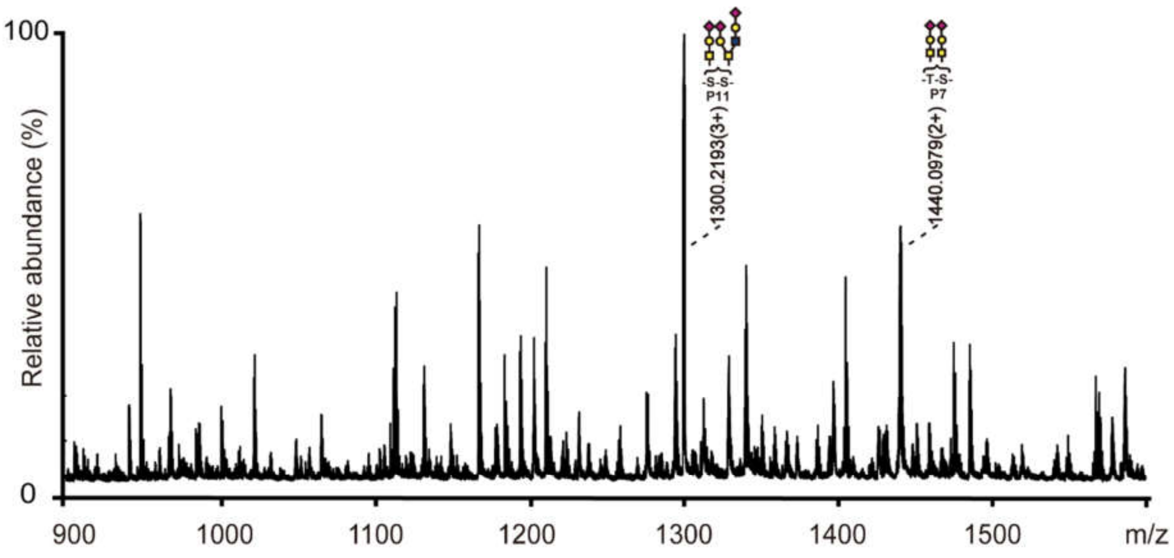

Figure S1. The further enrichment of the sample loading effluent in **Figure 1C** with HBS materials under optimal condition

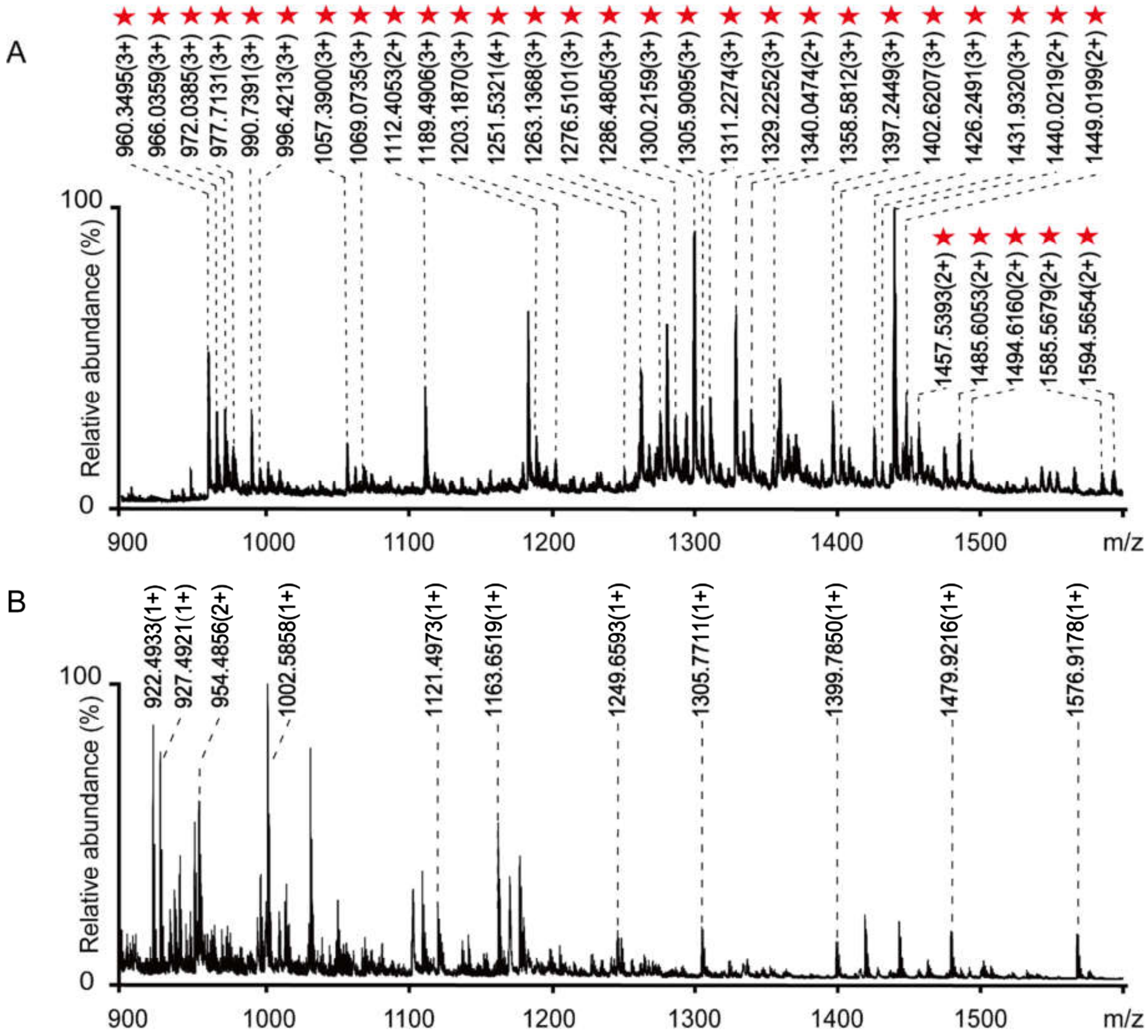

Figure S2. Mass spectra of the digests of bovine fetuin (10 μg) **(A)** after enrichment with HBS. **(B)** desalted digests mixture of bovine fetuin and BSA (1:20, w/w).

Measurement of Limit of Detection

To measure the limit of detection of the HBS enrichment method, a standard sialylated glycopeptide [m/z 1433.2025 (2+)] was used as a model sample due to the lack of standard O-glycopeptide. For this, 10 µl stocking solution (344 pmol/µl) of standard sialylated glycopeptide was diluted to 100 µl with 90 µl of 80% ACN (0.1% TFA). The resultant diluted standard sialylated glycopeptide solution was further diluted with ninefold volume of 80% ACN (0.1% TFA). In the same manner, different concentrations of standard sialylated glycopeptide were obtained. A series of diluted standard sialylated glycopeptide solution was loaded into 1 mg packed HBS GE-Loader, respectively, then enriched with HBS materials under optimized condition. The limit of detection was determined as the lowest concentration of standard sialylated glycopeptide with a signal-to-noise ratio greater than 3. The limit of detection of the HBS enrichment was 6.88 fmol/µl (**Figure S3**).

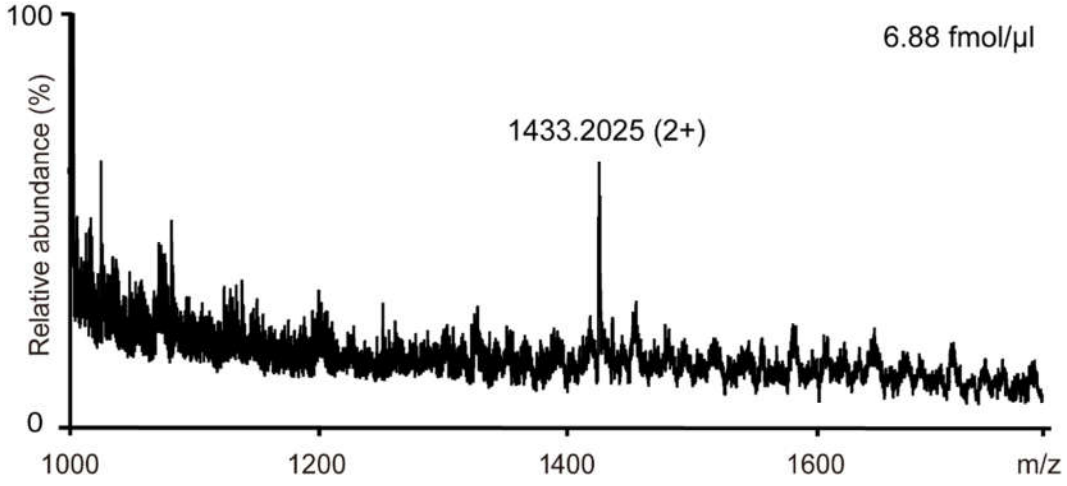

Figure S3. The limit of detection for standard sialylated glycopeptide on HBS.

Measurement of adsorption capacity

Due to the lack of O-glycopeptides standards, the O-glycopeptides were firstly enriched by ZIC-HILIC from bovine fetuin digests. The recovery of O-glycopeptides enriched with ZIC-HILIC was measured by using stable-isotope dimethyl labeling method (Boersema et al., 2009), and the recovery of typical two O-glycopeptides was over 84% (**Table S3**). Secondly, a series O-glycopeptides enriched from 20 µl (corresponding to 20 µg) bovine fetuin digests by ZIC-HILIC were dissolved in 80% ACN/0.1% TFA, then successively loaded into 1 mg HBS packed GE-Loader. Each sample loading effluent was collected and analyzed with Q-TOF MS. Before the point of the adsorption capacity, no O-glycopeptides signal could be detected in the mass spectra. When the amount of O-glycopeptides loaded into the HBS materials was beyond the adsorption capacity, O-glycopeptide signals could be detected. The adsorption capacity was determined before the point that O-glycopeptide signals were observed (**Figure S4**). Finally, the adsorption capacity of HBS (201.6 mg/g) was obtained by calculating the original bovine fetuin (240 µg) multiplication the recovery of ZIC-HILIC materials (84%).

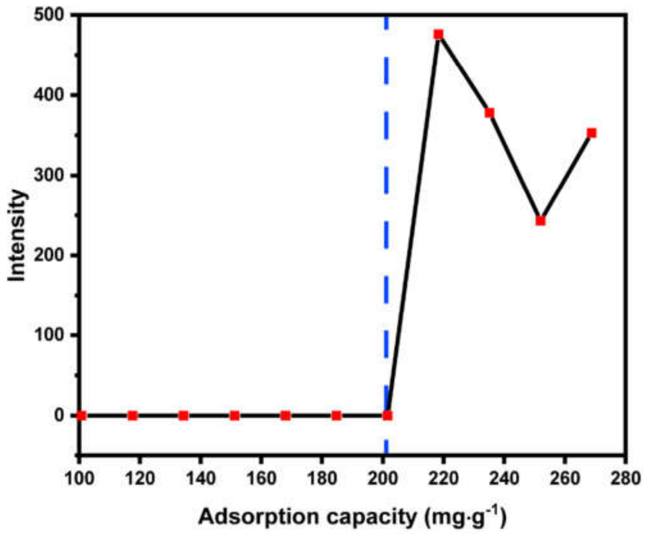

Figure S4. The adsorption capacity for bovine fetuin on HBS materials

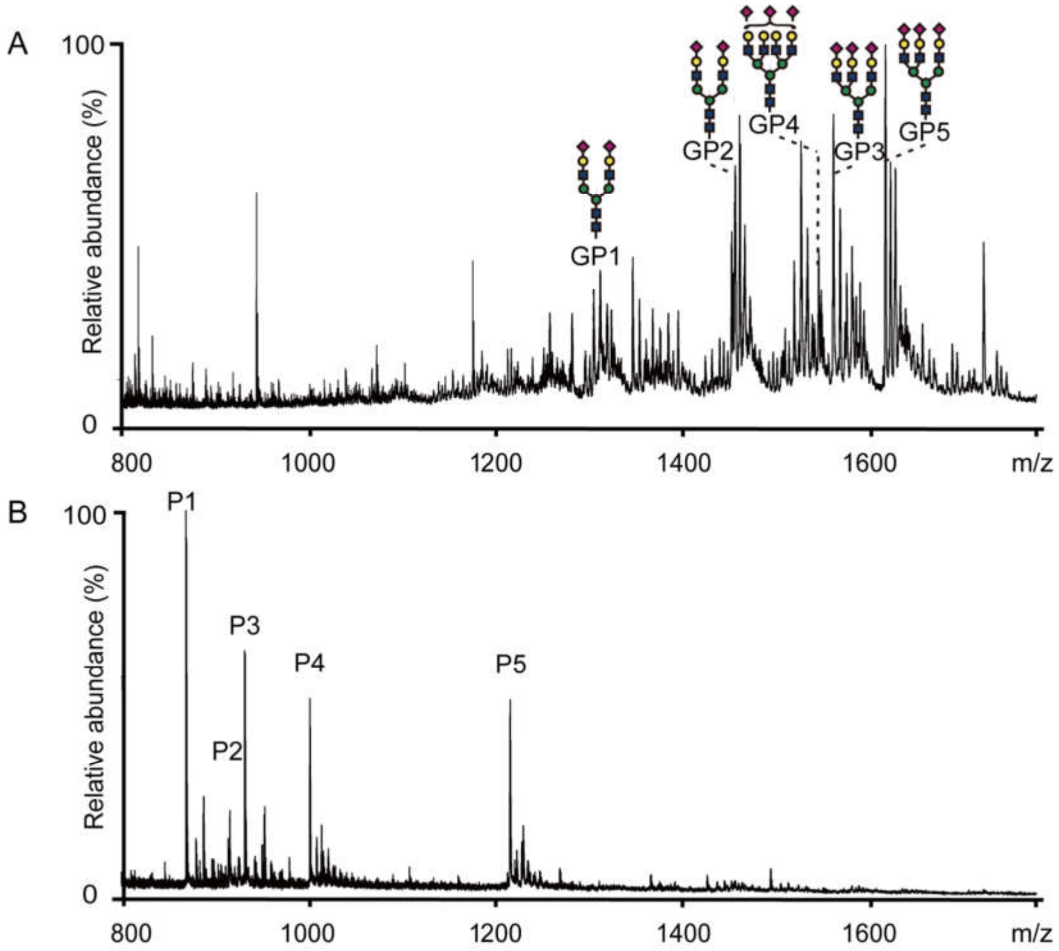

Figure S5. The verification of PNGase F efficiency by comparing the N-glycopeptides before (A) and after (B) PNGase F digestion from bovine fetuin tryptic disgests. The N-linked glycopeptides (GP) are marked with glycan structures and the deN-glycan peptides (P) are marked with the number. Glycopeptides are marked with their glycan structures: ■, GlcNAc; ●, mannose; ○, galactose; and ◆, N-acetylneuraminic acid (Neu5Ac). The detailed information of identified N-glycopeptides from bovine fetuin digest was shown in **Table S4**.

T48

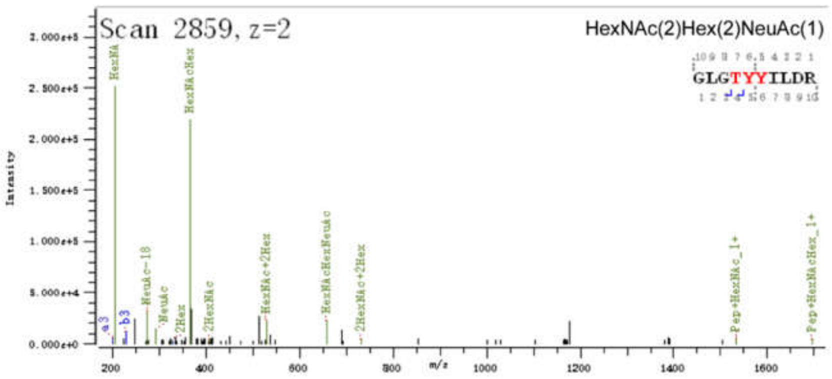

S104

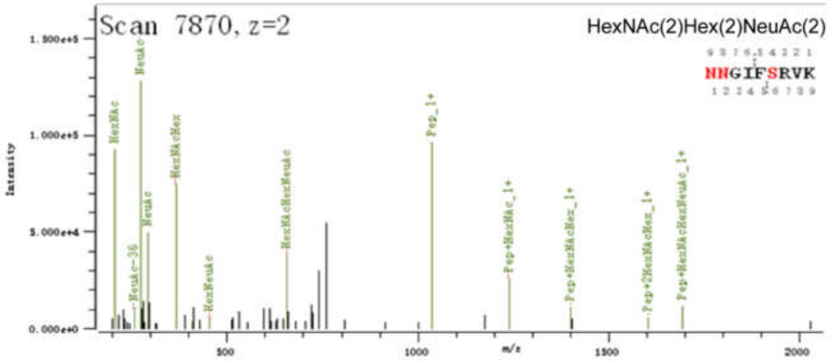

S134

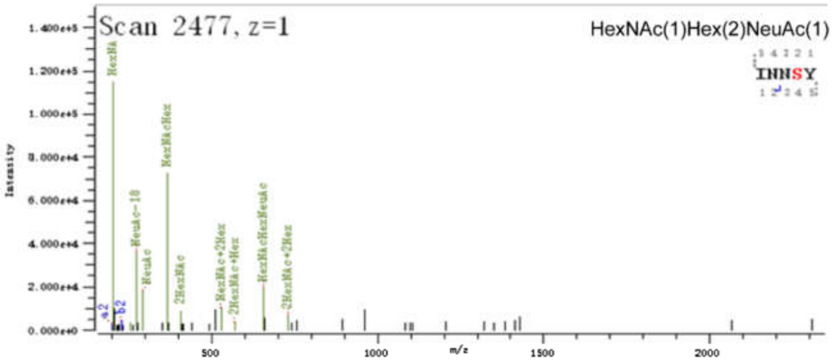

S173

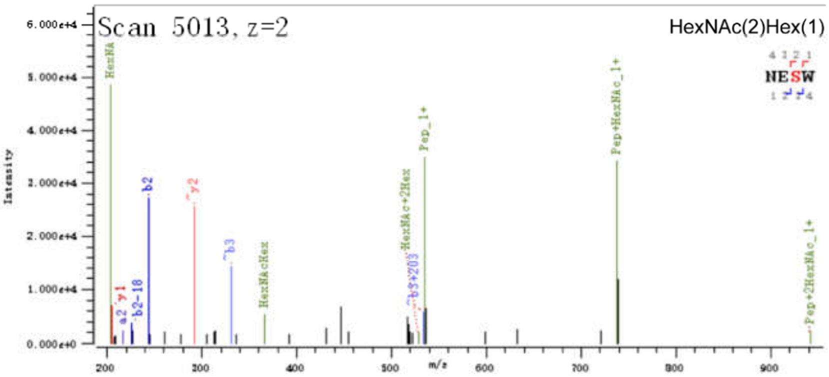

T301

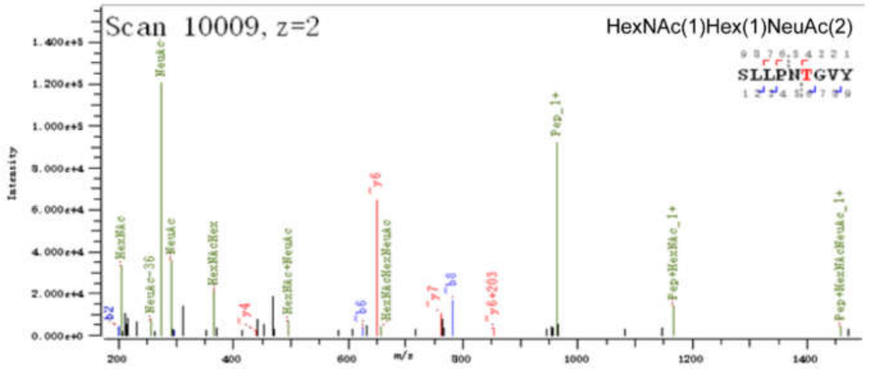

T316

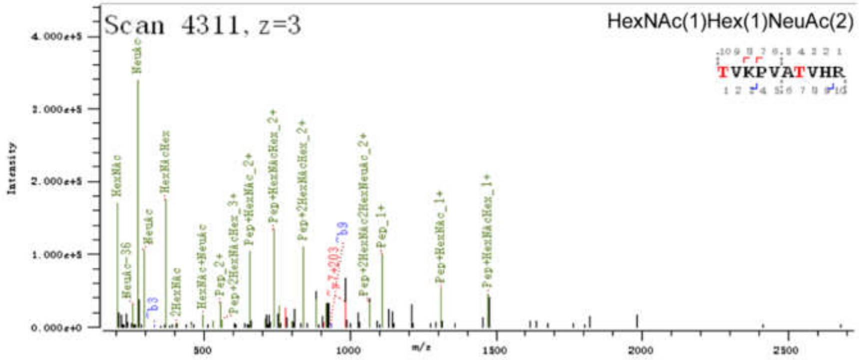

S340

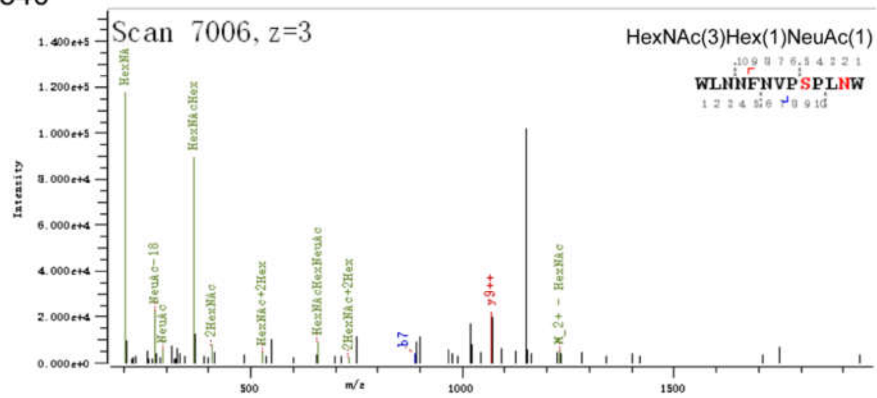

T435

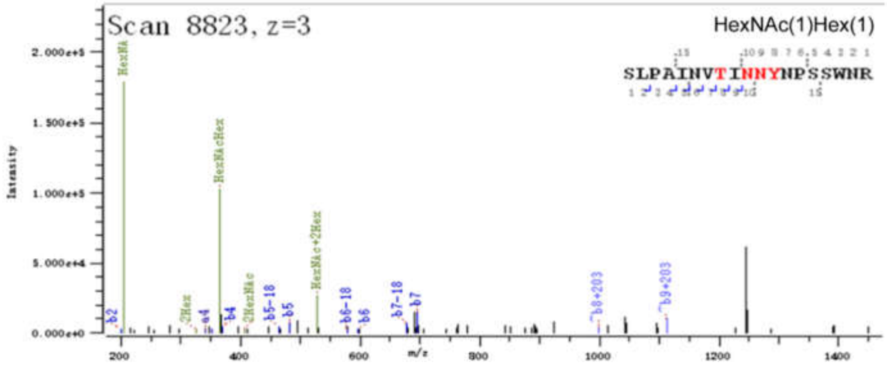

S459

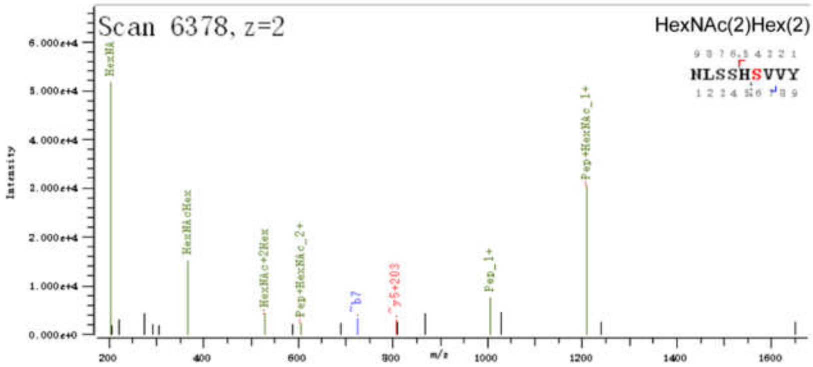

T499

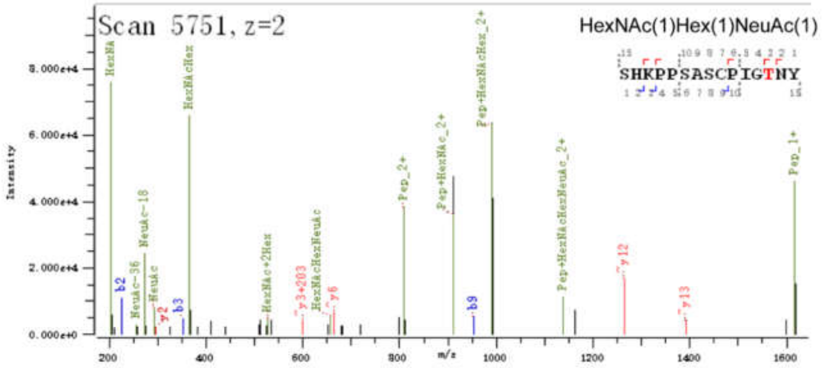

S538

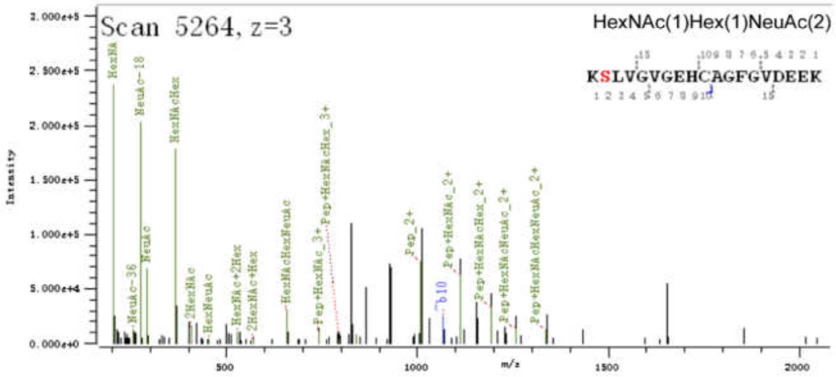

S562

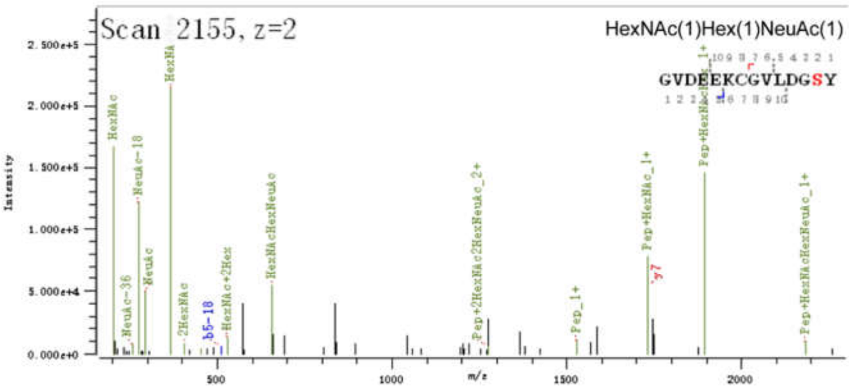

S578

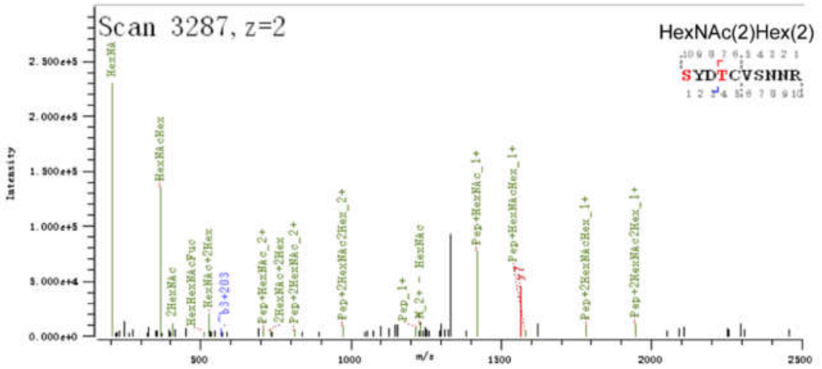

S584

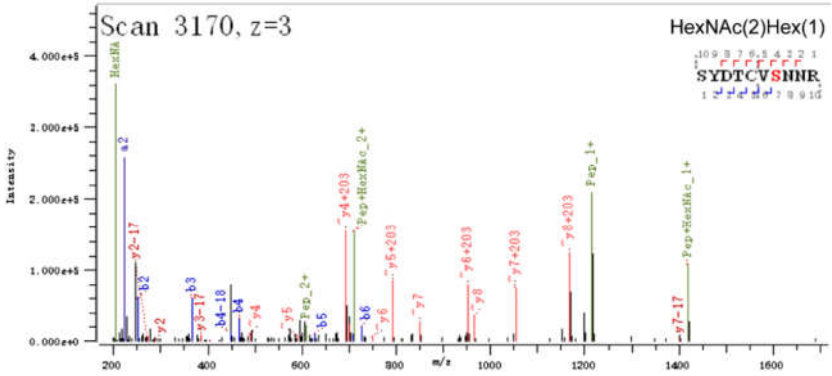

T665

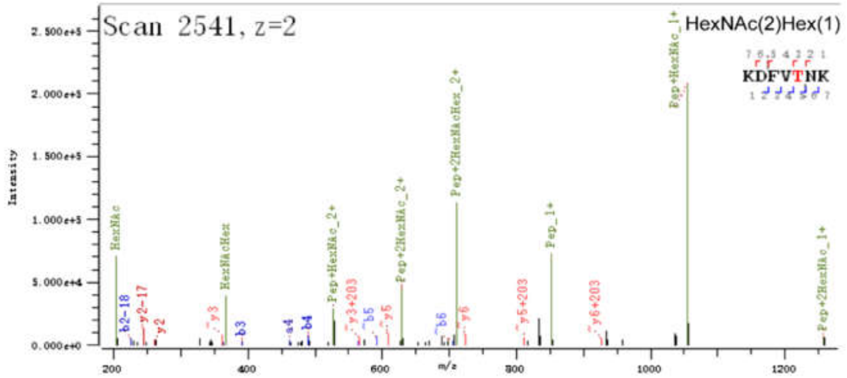

S680

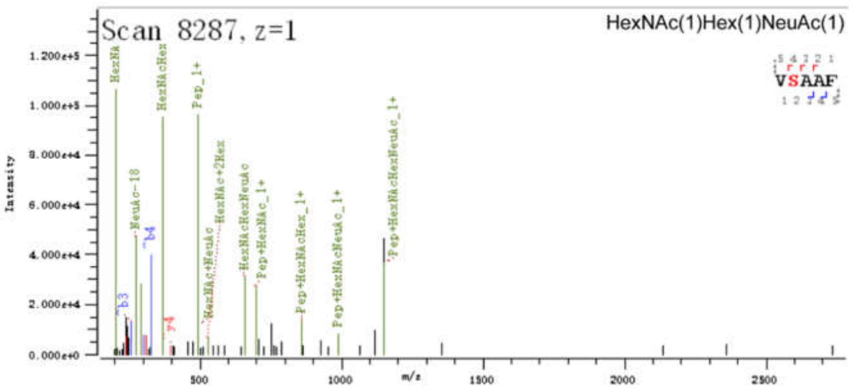

S741

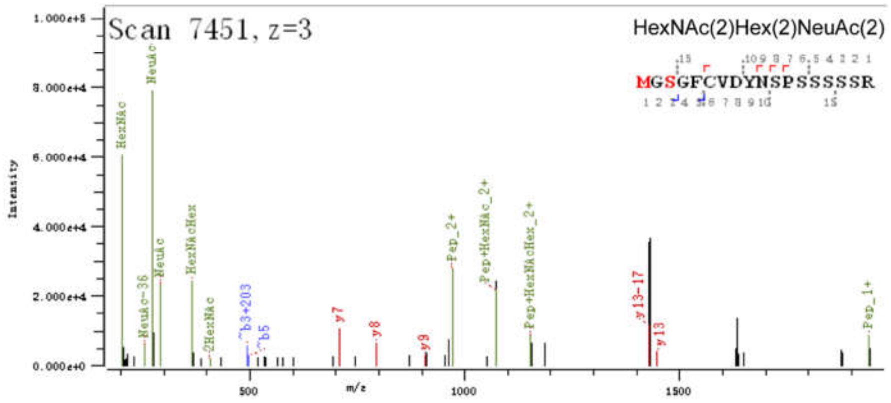

S749

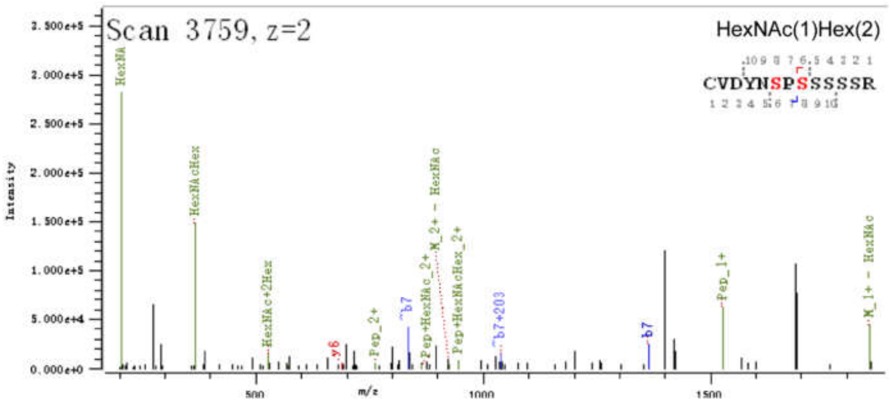

Figure S6. HCD MS/MS spectra of 18 unambiguous O-glycosylation sites identified using the Q-Exactive Orbitrap mass spectrometer. b and y product fragment ions are annotated in the peptide sequence.

Table S1. O-linked glycopeptides enriched with HBS from bovine fetuin PNGase F-elastase digests

NO.	Fetuin	Position	Peptide sequence	Observed (M+H)	Composition of glycans	Theoretical m/z	Experimental m/z
P1	Fetuin A	29-39	EPACDDPDTEQ	2223.7902	HexNAc(1)Hex(1)NeuAc(2)	1112.3987(2+)	1112.4053(2+)
P2	Fetuin A	170-180	TFNAESNGSYL	3205.2064	HexNAc(3)Hex(1)NeuAc(1)<br>HexNAc(2)Hex(1)NeuAc(1)	1069.0736(3+)	1069.0735(3+)
P3	Fetuin A	170-183	TFNAESNGSYLQLV	3787.3914	HexNAc(3)Hex(1)NeuAc(1)<br>HexNAc(2)Hex(2)NeuAc(1)	1263.1353(3+)	1263.1368(3+)
P4	Fetuin A	257-292	IPQPQPDGAEAEAPSAV<br>PDAAGPTPS	4073.7339	HexNAc(1)Hex(1)NeuAc(2)<br>HexNAc(1)Hex(1)NeuAc(1)	1358.5828(3+)	1358.5812(3+)
P5	Fetuin A	257-299	IPQPQPDGAEAEAPSAV<br>PDAAGPTPSAAGPPVA	5002.1842	HexNAc(2)Hex(2)NeuAc(2)<br>HexNAc(1)Hex(1)NeuAc(1)	1251.3015(4+)	1251.5321(4+)
P6	Fetuin A	262-282	EAEAPSAVPDAAGPTPS	3169.279	HexNAc(2)Hex(2)NeuAc(3)	1585.6395(2+)	1585.5679(2+)
P7	Fetuin A	266-282	EAEAPSAVPDAAGPTPS	2879.1944	HexNAc(1)Hex(1)NeuAc(1)<br>HexNAc(1)Hex(1)NeuAc(1)	960.4030(3+)	960.3495(3+)
P8	Fetuin A	266-282	EAEAPSAVPDAAGPTPS	2879.1944	HexNAc(1)Hex(1)NeuAc(1)<br>HexNAc(1)Hex(1)NeuAc(1)	966.0648(3+)<br>(NH_3)	966.0359 (3+)
P9	Fetuin A	266-282	EAEAPSAVPDAAGPTPS	2879.1944	HexNAc(1)Hex(1)NeuAc(1)<br>HexNAc(1)Hex(1)NeuAc(1)	1440.0979(2+)	1440.0219(2+)
P10	Fetuin A	266-282	EAEAPSAVPDAAGPTPS	2879.1944	HexNAc(1)Hex(1)NeuAc(1)<br>HexNAc(1)Hex(1)NeuAc(1)	1449.0972(2+)<br>(NH_3)	1449.0199(2+)
P11	Fetuin A	266-282	EAEAPSAVPDAAGPTPS	3170.2903	HexNAc(1)Hex(1)NeuAc(1)	1057.4350(3+)	1057.3900(3+)

					HexNAc(1)Hex(1)NeuAc(2)		
P12	Fetuin A	266-282	EAEAPSAVPDAAGPTPS	3170.2903	HexNAc(1)Hex(1)NeuAc(1) HexNAc(1)Hex(1)NeuAc(2)	1594.6452(2+)	1594.5654(2+)
P13	Fetuin A	267-288	AEAPSAVPDAAGPTPSA AGPPV	2259.7782	HexNAc(3)Hex(3)NeuAc(4)	1397.2422(3+)	1397.2449(3+)
P14	Fetuin A	268-282	EAPSAVPDAAGPTPS	2970.2072	HexNAc(1)Hex(1)NeuAc(2) HexNAc(1)Hex(1)NeuAc(1)	990.7406(3+)	990.7391(3+)
P15	Fetuin A	268-282	EAPSAVPDAAGPTPS	2970.2072	HexNAc(1)Hex(1)NeuAc(2) HexNAc(1)Hex(1)NeuAc(1)	996.4024(3+) (NH_3)	996.4213(3+)
P16	Fetuin A	268-282	EAPSAVPDAAGPTPS	2679.1122	HexNAc(1)Hex(1)NeuAc(1) HexNAc(1)Hex(1)NeuAc(1)	1340.0597(2+)	1340.0474(2+)
P17	Fetuin A	268-282	EAPSAVPDAAGPTPS	2970.2033	HexNAc(1)Hex(1)NeuAc(1) HexNAc(1)Hex(1)NeuAc(2)	1485.6053(2+)	1485.6053(2+)
P18	Fetuin A	268-282	EAPSAVPDAAGPTPS	2970.2033	HexNAc(1)Hex(1)NeuAc(1) HexNAc(1)Hex(1)NeuAc(2)	1494.6017(2+) (NH_3)	1494.6160(2+)
P19	Fetuin A	268-288	EAPSAVPDAAGPTPSAA GPPV	3827.6105	HexNAc(1)Hex(1)NeuAc(1) HexNAc(2)Hex(2)NeuAc(2)	1276.5417(3+)	1276.5101(3+)
P20	Fetuin A	268-289	EAPSAVPDAAGPTPSAA GPPVA	3607.5514	HexNAc(2)Hex(2)NeuAc(2) HexNAc(1)Hex(1)	1203.1887(3+)	1203.1870(3+)
P21	Fetuin A	268-289	EAPSAVPDAAGPTPSAA GPPVA	3898.6433	HexNAc(1)Hex(1)NeuAc(1) HexNAc(2)Hex(2)NeuAc(2)	1300.2193(3+)	1300.2159(3+)
P22	Fetuin A	268-289	EAPSAVPDAAGPTPSAA GPPVA	3898.6433	HexNAc(1)Hex(1)NeuAc(1) HexNAc(2)Hex(2)NeuAc(2)	1305.8811(3+)	1305.9095(3+)

						(NH$_3$)	
P23	Fetuin A	268-290	EAPSAVPDAAGPTPSAA GPPVAS	3985.6825	HexNAc(1)Hex(1)NeuAc(1) HexNAc(2)Hex(2)NeuAc(2)	1329.2324(3+)	1329.2252(2+)
P24	Fetuin A	271-282	SAVPDAAGPTPS	4276.6105	HexNAc(4)Hex(4)NeuAc(6)	1426.2035(3+)	1426.2491(3+)
P25	Fetuin A	271-282	SAVPDAAGPTPS	4276.6105	HexNAc(4)Hex(4)NeuAc(6)	1431.8702(3+) (NH$_3$)	1431.9320(3+)
P26	Fetuin A	319-331	ASVESSSGEAFHV	2261.7782	HexNAc(3)Hex(3)NeuAc(4)	1189.4542(3+)	1189.4906(3+)
P27	Fetuin A	319-331	ASVESSSGEAFHV	2551.8736	HexNAc(3)Hex(3)NeuAc(5)	1286.4860(3+)	1286.4805(3+)
P28	Fetuin A	319-331	ASVESSSGEAFHV	2621.9104	HexNAc(4)Hex(4)NeuAc(4)	1311.1649(3+)	1311.2274(3+)
P29	Fetuin A	319-332	ASVESSSGEAFHVG	2842.969	HexNAc(3)Hex(3)NeuAc(6)	1402.5249(3+)	1402.6207(3+)
P30	Fetuin B	283-296	KTEELQQQNTAPTN	2914.225	HexNAc(2)Hex(2)NeuAc(2)	972.0750(3+)	972.0385(3+)
P31	Fetuin B	283-296	KTEELQQQNTAPTN	2914.225	HexNAc(2)Hex(2)NeuAc(2)	977.7417(3+)	977.7131(3+)
P32	Fetuin B	283-296	KTEELQQQNTAPTN	2914.225	HexNAc(2)Hex(2)NeuAc(2)	1457.6125(2+)	1457.5393(2+)
*P33	Fetuin A	268-290	AEAPSAVPDAAGPTPSA AGPPVAS	2744.2453	HexNAc(1)Hex(1)NeuAc(1)	1372.6227(2+)	1372.7682(2+)

*: enriched with ZIC-HILIC materials

Table S2. Recovery of two O-glycopeptides from bovine fetuin digests after enrichment by HBS

Parellel	m/z=1340.0485(2+)		Recovery (light/heavy)	Parellel	m/z=1494.0962(2+)		Recovery (light/heavy)
	light labeled 1354.2106(2+)	heavy labeled 1356.2782(2+)			light labeled 1508.7896(2+)	heavy labeled 1510.8285(2+)	
1	860	901	95.4%	1	487	505	96.4%
2	997	1155	86.3%	2	510	514	99.2%
3	962	963	99.9%	3	507	536	94.6%
Average Recovery			93.9%±6.9%	Average Recovery			96.7%±2.3%

Table S3. Recovery of two O-glycopeptides from bovine fetuin digests after enrichmen by ZIC-HILIC

Parellel	m/z=1295.5308 (2+)		Recovery (light/heavy)	Parellel	m/z=1340.0485(2+)		Recovery (light/heavy)
	light labeled 1309.5583(2+)	heavy labeled 1311.5513(2+)			light labeled 1354.2106(2+)	heavy labeled 1356.2782(2+)	
1	474	556	85.2%	1	355	396	89.6%
2	272	310	87.7%	2	554	653	84.8%
3	771	963	80.1%	3	750	839	89.3%
Average Recovery			84.3%±3.9%	Average Recovery			87.9%±2.3%

Table S4. The information of typical N-glycopeptides of tryptic bovine fetuin before and after PNGase F digestion

No.	Peptide sequence	N-Glycopeptide m/z	deN-glycan pepitde m/z
P1	KLCPDCPLLAPLNDSR（Cys_CAM: 146, 149）	1315.9303(3+)	871.4247(2+)
P2	RPTGEVYDIEIDTLETTCHVLDPTPLANCSVR（Cys_CAM: 89, 100）	1470.2654(4+)	918.9318(4+)
P3	KLCPDCPLLAPLNDSR（Cys_CAM: 146, 149）	1577.5867(3+)	935.4619(2+)
P4	VVHAVEVALATFNAESN（176）GSYLQLVEISR	1561.6439(4+)	1006.5212(3+)
P5	RPTGEVYDIEIDTLETTCHVLDPTPLANCSVR（Cys_CAM: 89, 100）	1634.5735(4+)	1224.9293(3+)

Table S5. Identified O-linked glycopeptides and O-linked glycans in HKU1 S1 protein

No.	SequenceDot	ModificationsSummaryList	ModificationsResidueList	ModificationsProteinPositionList	Score	GlycanList
1	R.IsEyVVDVsYGLGTY.Y	S2(OGlycan/656.2276); Y4(Phospho/79.9663); S9(OGlycan/730.2644)	S,Y,S	36,38,43	41.29 - 41.29	HexNAc(1)Hex(1)NeuAc(1), HexNAc(2)Hex(2)
2	Y.GLGtyyILDR.V	T4(OGlycan/1021.3598); Y5(Phospho/79.9663); Y6(Phospho/79.9663)	T,Y,Y	48,49,50	166.27 - 166.27	HexNAc(2)Hex(2)NeuAc(1)
3	Y.LNttILFTGy.F	T3(OGlycan/1312.4552); T4(OGlycan/947.3230); Y10(Phospho/79.9663)	T,T,Y	59,60,66	42.10 - 42.10	HexNAc(1)Hex(1)NeuAc(2), HexNAc(2)Hex(2)NeuAc(2)
4	Y.LNttILFTGy.F	T3(OGlycan/859.3070); T4(OGlycan/1312.4552); Y10(Phospho/79.9663)	T,T,Y	59,60,66	47.00 - 47.00	HexNAc(2)Hex(1)NeuAc(1), HexNAc(2)Hex(2)NeuAc(2)
5	Y.LNttILFTGy.F	T3(OGlycan/947.3230); T4(OGlycan/1021.3598); Y10(Phospho/79.9663)	T,T,Y	59,60,66	30.24 - 30.24	HexNAc(1)Hex(1)NeuAc(2), HexNAc(2)Hex(2)NeuAc(1)
6	Y.LNttILFTGy.F	T3(OGlycan/947.3230); T4(OGlycan/1062.3864); Y10(Phospho/79.9663)	T,T,Y	59,60,66	32.82 - 32.82	HexNAc(1)Hex(1)NeuAc(2), HexNAc(3)Hex(1)NeuAc(1)
7	Y.LNttILFTGy.F	T3(OGlycan/947.3230); T4(OGlycan/1167.4177); Y10(Phospho/79.9663)	T,T,Y	59,60,66	46.42 - 46.42	HexNAc(1)Hex(1)NeuAc(2), HexNAc(2)Hex(2)Fuc(1)NeuAc(1)
8	Y.LNttILFTGy.F	T3(OGlycan/947.3230); T4(OGlycan/1312.4552); Y10(Phospho/79.9663)	T,T,Y	59,60,66	34.29 - 34.29	HexNAc(1)Hex(1)NeuAc(2), HexNAc(2)Hex(2)NeuAc(2)
9	R.VYLNttILF.T	T5(OGlycan/1167.4177); T6(OGlycan/714.2695)	T,T	59,60	115.56 - 349.02	HexNAc(2)Hex(2)Fuc(1)NeuAc(1),HexNAc(2)Hex(1)Fuc(1)
10	R.VYLNttILF.T	T5(OGlycan/730.2644); T6(OGlycan/714.2695)	T,T	59,60	155.29 - 192.26	HexNAc(2)Hex(2),HexNAc(2)Hex(1)Fuc(1)

11	K.GttYLSTLW.Y	T2(OGlycan/714.2695); S6(Phospho/79.9663); T7(Phospho/79.9663)	T,T	82,83	43.32 - 43.32	HexNAc(3)Hex(1)NeuAc(1), HexNAc(2)Hex(2)Fuc(1)NeuAc(1)
12	K.GttY.L	T2(OGlycan/1062.3864); T3(OGlycan/1167.4177)	T,T	82,83	49.99 - 49.99	HexNAc(3)Hex(1)NeuAc(1), HexNAc(2)Hex(2)NeuAc(2)
13	F.RDLSLKGttY.L	T2(OGlycan/1062.3864); T3(OGlycan/1312.4552)	T,T	82,83	60.05 - 60.05	HexNAc(1)Hex(2)HexNAc(1),Hex(2)NeuAc(1)
14	K.GttY.L	T8(OGlycan/818.2804); T9(OGlycan/527.1850)	T,T	82,83	64.96 - 73.20	HexNAc(2)Hex(2)NeuAc(1), HexNAc(2)Hex(2)NeuAc(2)
15	K.GttYLsTLW.Y	T2(OGlycan/1021.3598); T3(OGlycan/1312.4552)	T,T,S	82,83,86	41.44 - 41.44	HexNAc(2)Hex(2)NeuAc(2), HexNAc(1)Hex(2)NeuAc(1)
16	K.GtTYLstLW.Y	T2(OGlycan/818.2804); T3(OGlycan/1312.4552); S6(Phospho/79.9663)	T,S,T	82,86,87	187.08 - 187.08	HexNAc(2)Hex(1)Fuc(1)
17	F.RDLSLKGttY.L	T8(OGlycan/1312.4552); T9(OGlycan/1312.4552)	T,T	82,83	41.20 - 41.20	HexNAc(2)Hex(2)NeuAc(2), HexNAc(2)Hex(2)NeuAc(2)
18	Y.LsTLW.Y	S2(OGlycan/1312.4552)	S	86	375.19 - 375.19	HexNAc(2)Hex(2)NeuAc(2)
19	F.nnGIFsRVK.N	N1(Deamidated/0.9840); N2(Deamidated/0.9840); S6(OGlycan/1312.4552)	N,N,S	99,100,104	120.25 - 120.25	HexNAc(2)Hex(2)NeuAc(2)
20	F.stIVIGSVF.I	S1(OGlycan/1312.4552); T2(OGlycan/1312.4552)	S,T	122,123	33.33 - 33.33	HexNAc(2)Hex(2)NeuAc(2), HexNAc(2)Hex(2)NeuAc(2)
21	F.INNsY.T	S4(OGlycan/818.2804)	S	134	152.65 - 152.65	HexNAc(1)Hex(2)NeuAc(1)
22	Y.TMCEYPHtICKSKGSSR.N	T8(OGlycan/656.2276)	T	161	36.83 - 36.83	HexNAc(1)Hex(1)NeuAc(1)
23	R.NEsW.H	S3(OGlycan/568.2116)	S	173	327.69 - 374.45	HexNAc(2)Hex(1)
24	R.NEsW.H	S3(OGlycan/730.2644)	S	173	350.70 - 376.86	HexNAc(2)Hex(2)
25	K.nFtYNVsTDW.L	N1(Deamidated/0.9840); T3(OGlycan/365.1322); S7(OGlycan/1167.4177)	N,T,S	188,190,194	86.49 - 86.49	HexNAc(1)Hex(1),HexNAc(2)Hex(2)Fuc(1)NeuAc(1)
26	K.nFtYnVsTDW.L	N1(Deamidated/0.9840); T3(OGlycan/1021.3598);	N,T,N,S	188,190,192, 194	71.87 - 71.87	HexNAc(2)Hex(2),HexNAc(2)Hex(2)NeuAc(1)

		N5(Deamidated/0.9840); S7(OGlycan/730.2644)				
27	F.tyNVSTDWLY.F	T1(OGlycan/1021.3598); Y2(Phospho/79.9663)	T,Y	190,191	128.19 - 128.19	HexNAc(2)Hex(2)NeuAc(1)
28	F.tyNVSTDWLY.F	T1(OGlycan/568.2116); Y2(Phospho/79.9663)	T,Y	190,191	63.70 - 63.70	HexNAc(2)Hex(1)
29	F.tyNVsTDWLY.F	T1(OGlycan/656.2276); Y2(Phospho/79.9663); S5(OGlycan/1167.4177)	T,Y,S	190,191,194	33.86 - 33.86	HexNAc(1)Hex(1)NeuAc(1), HexNAc(2)Hex(2)Fuc(1)NeuAc(1)
30	F.tyNVSTDWLY.F	T1(OGlycan/859.3070); Y2(Phospho/79.9663)	T,Y	190,191	116.38 - 116.38	HexNAc(2)Hex(1)NeuAc(1)
31	F.tYNVSTDWLY.F	T1(OGlycan/1312.4552)	T	190	244.34 - 244.34	HexNAc(2)Hex(2)NeuAc(2)
32	F.tYNVSTDWLY.F	T1(OGlycan/947.3230)	T	190	246.10 - 326.94	HexNAc(1)Hex(1)NeuAc(2)
33	F.tYNVSTDWLy.F	T1(OGlycan/818.2804); Y10(Phospho/79.9663)	T,Y	190,199	74.27 - 74.27	HexNAc(1)Hex(2)NeuAc(1)
34	F.TyNVstDWLY.F	Y2(Phospho/79.9663); S5(OGlycan/365.1322); T6(OGlycan/730.2644)	Y,S,T	191,194,195	33.26 - 33.26	HexNAc(1)Hex(1),HexNAc(2)Hex(2)
35	F.TYNVstDWLy.F	S5(OGlycan/656.2276); T6(OGlycan/730.2644); Y10(Phospho/79.9663)	S,T,Y	194,195,199	113.28 - 113.28	HexNAc(1)Hex(1)NeuAc(1), HexNAc(2)Hex(2)
36	Y.AYYADsGMPTtF.L	S6(OGlycan/947.3230); T11(Phospho/79.9663)	S,T	216,221	125.67 - 125.67	HexNAc(1)Hex(1)NeuAc(2)
37	Y.YADsGmPtTF.L	S4(OGlycan/730.2644); M6(Oxidation/15.9949); T8(OGlycan/818.2804)	S,M,T	216,218,220	61.74 - 61.74	HexNAc(2)Hex(2),HexNAc(1)Hex(2)NeuAc(1)
38	Y.ADSGMPttF.L	T7(OGlycan/1312.4552); T8(OGlycan/1312.4552)	T,T	220,221	19.90 - 19.90	HexNAc(2)Hex(2)NeuAc(2), HexNAc(2)Hex(2)NeuAc(2)
39	Y.YVLPLtCNAIssnTDNETLQY.W	T6(Phospho/79.9663); S11(Phospho/79.9663); S12(OGlycan/1021.3598); N13(Deamidated/0.9840)	T,S,S,N	241,246,247,248	51.31 - 51.31	HexNAc(2)Hex(2)NeuAc(1)
40	K.FDNRGVItnAVDCSSSF.F	T8(OGlycan/1021.3598); N9(Deamidated/0.9840)	T,N	277,278	124.36 - 124.36	HexNAc(2)Hex(2)NeuAc(1)

41	R.GVItNAVDCSSS F.F	T4(OGlycan/1021.3598)	T	277	85.87 - 105.22	HexNAc(2)Hex(2)NeuAc(1)
42	F.DNRGVItNAVDC SSSF.F	T7(OGlycan/1021.3598)	T	277	38.48 - 38.48	HexNAc(2)Hex(2)NeuAc(1)
43	R.GVItNAVDCSSS F.F	T4(OGlycan/947.3230)	T	277	100.50 - 225.44	HexNAc(1)Hex(1)NeuAc(2)
44	R.GVITNAVDCssS F.F	S10(OGlycan/656.2276); S11(OGlycan/568.2116)	S,S	283,284	63.54 - 63.54	HexNAc(2)Hex(1),HexNAc(1)Hex(1)NeuAc(1)
45	K.sLLPNtGVYDLS GFTVKPVATVHR. R	S1(OGlycan/1312.4552); T6(OGlycan/947.3230)	S,T	296,301	34.68 - 121.34	HexNAc(1)Hex(1)NeuAc(2), HexNAc(2)Hex(2)NeuAc(2)
46	K.sLLPNTGVY.D	S1(OGlycan/1167.4177)	S	296	268.13 - 284.05	HexNAc(2)Hex(2)Fuc(1)Neu Ac(1)
47	K.sLLPNtGVYDLS GF.T	S1(OGlycan/511.1901); T6(OGlycan/818.2804)	S,T	296,301	40.34 - 40.34	HexNAc(1)Hex(1)Fuc(1),He xNAc(1)Hex(2)NeuAc(1)
48	K.sLLPNTGVY.D	S1(OGlycan/1312.4552)	S	296	228.05 - 394.30	HexNAc(2)Hex(2)NeuAc(2)
49	K.sLLPNTGVY.D	S1(OGlycan/947.3230)	S	296	285.46 - 373.51	HexNAc(1)Hex(1)NeuAc(2)
50	K.SLLPNtGVY.D	T6(OGlycan/1312.4552)	T	301	343.40 - 343.40	HexNAc(2)Hex(2)NeuAc(2)
51	K.SLLPNtGVY.D	T6(OGlycan/947.3230)	T	301	406.41 - 406.41	HexNAc(1)Hex(1)NeuAc(2)
52	Y.DLsGFtVKPVAT VHR.R	S3(OGlycan/947.3230); T6(OGlycan/1167.4177)	S,T	307,310	109.44 - 204.11	HexNAc(1)Hex(1)NeuAc(2), HexNAc(2)Hex(2)Fuc(1)Neu Ac(1)
53	Y.DLsGFtVKPVAT VHR.R	S3(OGlycan/1062.3864); T6(OGlycan/947.3230)	S,T	307,310	33.85 - 33.85	HexNAc(1)Hex(1)NeuAc(2), HexNAc(3)Hex(1)NeuAc(1)
54	Y.DLsGFtVKPVAT VHR.R	S3(OGlycan/656.2276); T6(OGlycan/1312.4552)	S,T	307,310	155.17 - 252.99	HexNAc(1)Hex(1)NeuAc(1), HexNAc(2)Hex(2)NeuAc(2)
55	Y.DLsGFtVKPVAT VHR.R	S3(OGlycan/1312.4552); T6(OGlycan/656.2276)	S,T	307,310	31.50 - 134.70	HexNAc(1)Hex(1)NeuAc(1), HexNAc(2)Hex(2)NeuAc(2)
56	Y.DLsGFtVKPVAT VHR.R	S3(OGlycan/1312.4552); T6(OGlycan/1312.4552)	S,T	307,310	60.96 - 149.10	HexNAc(2)Hex(2)NeuAc(2), HexNAc(2)Hex(2)NeuAc(2)
57	Y.DLsGFtVKPVAT VHR.R	S3(OGlycan/1312.4552); T6(OGlycan/1021.3598)	S,T	307,310	19.97 - 38.48	HexNAc(2)Hex(2)NeuAc(1), HexNAc(2)Hex(2)NeuAc(2)

58	Y.DLsGFtVKPVATVHR.R	S3(OGlycan/656.2276); T6(OGlycan/947.3230)	S,T	307,310	147.38 - 147.38	HexNAc(1)Hex(1)NeuAc(1), HexNAc(1)Hex(1)NeuAc(2)
59	Y.DLsGFtVKPVATVHR.R	S3(OGlycan/1312.4552); T6(OGlycan/947.3230)	S,T	307,310	26.44 - 134.44	HexNAc(1)Hex(1)NeuAc(2), HexNAc(2)Hex(2)NeuAc(2)
60	Y.DLSGFtVKPVAtVHR.R	T6(OGlycan/1021.3598); T12(OGlycan/947.3230)	T,T	310,316	172.84 - 399.36	HexNAc(1)Hex(1)NeuAc(2), HexNAc(2)Hex(2)NeuAc(1)
61	Y.DLSGFtVKPVAtVHR.R	T6(OGlycan/1312.4552); T12(OGlycan/656.2276)	T,T	310,316	207.00 - 211.37	HexNAc(1)Hex(1)NeuAc(1), HexNAc(2)Hex(2)NeuAc(2)
62	Y.DLSGFtVKPVAtVHR.R	T6(OGlycan/947.3230); T12(OGlycan/947.3230)	T,T	310,316	132.26 - 486.77	HexNAc(1)Hex(1)NeuAc(2), HexNAc(1)Hex(1)NeuAc(2)
63	Y.DLSGFtVKPVAtVHR.R	T6(OGlycan/1021.3598); T12(OGlycan/1312.4552)	T,T	310,316	214.60 - 278.12	HexNAc(2)Hex(2)NeuAc(1), HexNAc(2)Hex(2)NeuAc(2)
64	Y.DLSGFtVKPVAtVHR.R	T6(OGlycan/947.3230); T12(OGlycan/1312.4552)	T,T	310,316	56.18 - 527.82	HexNAc(1)Hex(1)NeuAc(2), HexNAc(2)Hex(2)NeuAc(2)
65	Y.DLSGFtVKPVAtVHR.R	T6(OGlycan/947.3230); T12(OGlycan/656.2276)	T,T	310,316	92.78 - 305.16	HexNAc(1)Hex(1)NeuAc(1), HexNAc(1)Hex(1)NeuAc(2)
66	F.tVKPVAtVHR.R	T1(OGlycan/947.3230); T7(OGlycan/1312.4552)	T,T	310,316	229.51 - 286.45	HexNAc(1)Hex(1)NeuAc(2), HexNAc(2)Hex(2)NeuAc(2)
67	Y.DLSGFtVKPVAtVHR.R	T6(OGlycan/1312.4552); T12(OGlycan/1312.4552)	T,T	310,316	88.43 - 251.41	HexNAc(2)Hex(2)NeuAc(2), HexNAc(2)Hex(2)NeuAc(2)
68	Y.DLSGFtVKPVAtVHR.R	T6(OGlycan/947.3230); T12(OGlycan/1167.4177)	T,T	310,316	103.04 - 103.04	HexNAc(1)Hex(1)NeuAc(2), HexNAc(2)Hex(2)Fuc(1)NeuAc(1)
69	F.tVKPVAtVHR.R	T1(OGlycan/947.3230); T7(OGlycan/947.3230)	T,T	310,316	364.80 - 442.50	HexNAc(1)Hex(1)NeuAc(2), HexNAc(1)Hex(1)NeuAc(2)
70	K.WLNNFNVPsPLnW.E	S9(OGlycan/1062.3864); N12(Deamidated/0.9840)	S,N	340,343	59.84 - 59.84	HexNAc(3)Hex(1)NeuAc(1)
71	F.NLstLLR.L	S3(OGlycan/527.1850); T4(OGlycan/1312.4552)	S,T	357,358	157.85 - 157.85	HexNAc(2)Hex(2)NeuAc(2), HexNAc(1)Hex(2)
72	F.NLStLLRLVHTDsFsCNNF.D	T4(OGlycan/818.2804); S13(Phospho/79.9663); S15(Phospho/79.9663)	T,S,S	358,367,369	43.71 - 43.71	HexNAc(1)Hex(2)NeuAc(1)
73	R.LVHtDsFSCNNF.D	T4(OGlycan/494.1748); S6(OGlycan/714.2695)	T,S	365,367	61.68 - 61.68	HexNAc(2)Hex(1)Fuc(1),HexNAc(1)NeuAc(1)

74	R.LVHtDsFSCNNF.D	T4(OGlycan/527.1850); S6(OGlycan/494.1748)	T,S	365,367	147.74 - 147.74	HexNAc(1)Hex(2),HexNAc(1)NeuAc(1)
75	R.LVHtDSFSCNNF.D	T4(OGlycan/947.3230)	T	365	77.26 - 160.14	HexNAc(1)Hex(1)NeuAc(2)
76	F.NLSTLLRLVHtDsFsCNNF.D	T11(OGlycan/818.2804); S13(Phospho/79.9663); S15(Phospho/79.9663)	T,S,S	365,367,369	65.00 - 65.00	HexNAc(1)Hex(2)NeuAc(1)
77	R.LVHtDSFSCNNF.D	T4(OGlycan/656.2276)	T	365	95.47 - 104.01	HexNAc(1)Hex(1)NeuAc(1)
78	R.LVHTDsFsCNNF.D	S6(OGlycan/859.3070); S8(OGlycan/818.2804)	S,S	367,369	104.80 - 104.80	HexNAc(2)Hex(1)NeuAc(1), HexNAc(1)Hex(2)NeuAc(1)
79	R.LVHTDsFsCNNF.D	S6(OGlycan/494.1748); S8(OGlycan/527.1850)	S,S	367,369	176.24 - 176.24	HexNAc(1)Hex(2),HexNAc(1)NeuAc(1)
80	R.LVHTDSFsCNNF.D	S8(OGlycan/1021.3598)	S	369	49.42 - 49.42	HexNAc(2)Hex(2)NeuAc(1)
81	R.sDLQLGSSGF.L	S1(OGlycan/947.3230)	S	399	317.42 - 425.27	HexNAc(1)Hex(1)NeuAc(2)
82	R.SDLQLGsSGF.L	S7(OGlycan/947.3230)	S	405	407.56 - 407.56	HexNAc(1)Hex(1)NeuAc(2)
83	R.SDLQLGSsGFLQSSNY.K	S8(OGlycan/1021.3598)	S	406	108.75 - 108.75	HexNAc(2)Hex(2)NeuAc(1)
84	R.SDLQLGSsGFLQSSNy.K	S8(OGlycan/947.3230); Y16(Phospho/79.9663)	S,Y	406,414	99.54 - 99.54	HexNAc(1)Hex(1)NeuAc(2)
85	F.LQssNyK.I	S3(OGlycan/527.1850); S4(OGlycan/494.1748); Y6(Phospho/79.9663)	S,S,Y	411,412,414	173.51 - 173.51	HexNAc(1)Hex(2),HexNAc(1)NeuAc(1)
86	F.LQssNyK.I	S3(OGlycan/818.2804); S4(OGlycan/714.2695); Y6(Phospho/79.9663)	S,S,Y	411,412,414	144.69 - 144.69	HexNAc(2)Hex(1)Fuc(1),HexNAc(1)Hex(2)NeuAc(1)
87	F.LQssNyK.I	S3(OGlycan/365.1322); S4(OGlycan/1312.4552); Y6(Phospho/79.9663)	S,S,Y	411,412,414	120.96 - 120.96	HexNAc(1)Hex(1),HexNAc(2)Hex(2)NeuAc(2)
88	F.LQssNYKIDTTsSSCQLY.Y	S3(Phospho/79.9663); S4(Phospho/79.9663); S12(OGlycan/1021.3598)	S,S,S	411,412,420	49.80 - 49.80	HexNAc(2)Hex(2)NeuAc(1)

89	F.LQsSNY.K	S3(OGlycan/947.3230)	S	411	435.27 - 450.78	HexNAc(1)Hex(1)NeuAc(2)
90	F.LQSsNY.K	S4(OGlycan/947.3230)	S	412	469.88 - 469.88	HexNAc(1)Hex(1)NeuAc(2)
91	Y.KIDtTSSSCQLY.Y	T4(OGlycan/494.1748)	T	418	237.74 - 427.49	HexNAc(1)NeuAc(1)
92	Y.KIDtTSSSCQLYY.S	T4(OGlycan/494.1748)	T	418	201.81 - 201.81	HexNAc(1)NeuAc(1)
93	Y.KIDtTSSSCQLYY.S	T4(OGlycan/656.2276)	T	418	129.04 - 218.39	HexNAc(1)Hex(1)NeuAc(1)
94	K.IDTtSSSCQLY.Y	T4(OGlycan/947.3230)	T	419	53.07 - 286.97	HexNAc(1)Hex(1)NeuAc(2)
95	Y.KIDTtSSSCQLY.Y	T5(OGlycan/1312.4552)	T	419	233.63 - 233.63	HexNAc(2)Hex(2)NeuAc(2)
96	Y.KIDTtSSSCQLYY.S	T5(OGlycan/1312.4552)	T	419	221.57 - 244.02	HexNAc(2)Hex(2)NeuAc(2)
97	K.IDTtsSSCQLYY.S	T4(OGlycan/1312.4552); S5(OGlycan/1312.4552)	T,S	419,420	91.96 - 91.96	HexNAc(2)Hex(2)NeuAc(2), HexNAc(2)Hex(2)NeuAc(2)
98	K.IDTTsSSCQLY.Y	S5(OGlycan/1312.4552)	S	420	167.41 - 325.40	HexNAc(2)Hex(2)NeuAc(2)
99	K.IDTTsSSCQLY.Y	S5(OGlycan/947.3230)	S	420	248.85 - 661.05	HexNAc(1)Hex(1)NeuAc(2)
100	Y.KIDTTsSSCQLY.Y	S6(OGlycan/1021.3598)	S	420	330.54 - 330.54	HexNAc(2)Hex(2)NeuAc(1)
101	Y.KIDTTsSSCQLYY.S	S6(OGlycan/1312.4552)	S	420	228.68 - 228.68	HexNAc(2)Hex(2)NeuAc(2)
102	Y.KIDTTsSSCQLY.Y	S6(OGlycan/1312.4552)	S	420	208.09 - 339.94	HexNAc(2)Hex(2)NeuAc(2)
103	Y.KIDTTsSSCQLY.Y	S6(OGlycan/947.3230)	S	420	456.99 - 498.89	HexNAc(1)Hex(1)NeuAc(2)
104	K.IDTTSsSCQLY.Y	S6(OGlycan/947.3230)	S	421	564.21 - 564.21	HexNAc(1)Hex(1)NeuAc(2)
105	Y.KIDTTSsSCQLY.Y	S7(OGlycan/1021.3598)	S	421	154.48 - 313.40	HexNAc(2)Hex(2)NeuAc(1)

106	Y.KIDTTSsSCQLY.Y	S7(OGlycan/859.3070)	S	421	164.69 - 164.69	HexNAc(2)Hex(1)NeuAc(1)
107	Y.KIDTTSsSCQLY.Y	S7(OGlycan/947.3230)	S	421	305.90 - 305.90	HexNAc(1)Hex(1)NeuAc(2)
108	K.IDTTSSsCQLYY.S	S7(OGlycan/947.3230)	S	422	159.51 - 235.68	HexNAc(1)Hex(1)NeuAc(2)
109	Y.KIDTTSSsCQLY.Y	S8(OGlycan/1021.3598)	S	422	252.16 - 397.31	HexNAc(2)Hex(2)NeuAc(1)
110	Y.KIDTTSSsCQLY.Y	S8(OGlycan/859.3070)	S	422	270.05 - 270.05	HexNAc(2)Hex(1)NeuAc(1)
111	Y.KIDTTSSsCQLYY.S	S8(OGlycan/947.3230)	S	422	159.70 - 431.53	HexNAc(1)Hex(1)NeuAc(2)
112	K.IDTTSSsCQLY.Y	S7(OGlycan/1312.4552)	S	422	152.18 - 373.58	HexNAc(2)Hex(2)NeuAc(2)
113	K.IDTTSSsCQLY.Y	S7(OGlycan/1167.4177)	S	422	161.64 - 258.74	HexNAc(2)Hex(2)Fuc(1)NeuAc(1)
114	Y.KIDTTSSsCQLYY.S	S8(OGlycan/1021.3598)	S	422	129.81 - 193.28	HexNAc(2)Hex(2)NeuAc(1)
115	Y.sLPAINVtINNY.N	S1(OGlycan/714.2695); T8(OGlycan/730.2644)	S,T	428,435	56.48 - 202.91	HexNAc(2)Hex(2),HexNAc(2)Hex(1)Fuc(1)
116	Y.YsLPAINVtINNY.N	S2(OGlycan/730.2644); T9(OGlycan/818.2804)	S,T	428,435	150.78 - 150.78	HexNAc(2)Hex(2),HexNAc(1)Hex(2)NeuAc(1)
117	Y.YsLPAINVtINNY.N	S2(OGlycan/730.2644); T9(OGlycan/1167.4177)	S,T	428,435	61.06 - 100.26	HexNAc(2)Hex(2),HexNAc(2)Hex(2)Fuc(1)NeuAc(1)
118	Y.YsLPAINVtINNY.N	S2(OGlycan/527.1850); T9(OGlycan/1021.3598)	S,T	428,435	61.59 - 61.59	HexNAc(2)Hex(2)NeuAc(1), HexNAc(1)Hex(2)
119	Y.sLPAINVtINNY.N	S1(OGlycan/714.2695); T8(OGlycan/1167.4177)	S,T	428,435	167.83 - 189.27	HexNAc(2)Hex(2)Fuc(1)NeuAc(1),HexNAc(2)Hex(1)Fuc(1)
120	Y.sLPAINVtInNY.N	S1(OGlycan/494.1748); T8(OGlycan/947.3230); N10(Deamidated/0.9840)	S,T,N	428,435,437	155.40 - 170.57	HexNAc(1)Hex(1)NeuAc(2), HexNAc(1)NeuAc(1)
121	Y.SLPAINVtInnyNPSSWNR.R	T8(OGlycan/365.1322); N10(Deamidated/0.9840); N11(Deamidated/0.9840); Y12(Phospho/79.9663)	T,N,N,Y	435,437,438,439	140.25 - 140.25	HexNAc(1)Hex(1)

122	Y.SLPAINVtInNynPSSWNR.R	T8(OGlycan/365.1322); N10(Deamidated/0.9840); Y12(Phospho/79.9663); N13(Deamidated/0.9840)	T,N,Y,N	435,437,439,440	209.54 - 209.54	HexNAc(1)Hex(1)
123	Y.SLPAINVtInnyNPSSWNR.R	T8(OGlycan/527.1850); N10(Deamidated/0.9840); N11(Deamidated/0.9840); Y12(Phospho/79.9663)	T,N,N,Y	435,437,438,439	142.51 - 142.51	HexNAc(1)Hex(2)
124	Y.SLPAINVtInNyNPsSWnR.R	T8(OGlycan/568.2116); N10(Deamidated/0.9840); Y12(Phospho/79.9663); S15(OGlycan/730.2644); N18(Deamidated/0.9840)	T,N,Y,S,N	435,437,439,442,445	184.68 - 184.68	HexNAc(2)Hex(1),HexNAc(2)Hex(2)
125	Y.SLPAINVtINNYNPsSW.N	T8(OGlycan/818.2804); S15(OGlycan/947.3230)	T,S	435,442	170.94 - 170.94	HexNAc(1)Hex(1)NeuAc(2), HexNAc(1)Hex(2)NeuAc(1)
126	Y.SLPAINVtINNynPsSWnR.R	T8(OGlycan/568.2116); Y12(Phospho/79.9663); N13(Deamidated/0.9840); S15(OGlycan/568.2116); N18(Deamidated/0.9840)	T,Y,N,S,N	435,439,440,442,445	64.17 - 64.17	HexNAc(2)Hex(1),HexNAc(2)Hex(1)
127	Y.SLPAINVtInNyNPSSWnR.R	T8(OGlycan/527.1850); N10(Deamidated/0.9840); Y12(Phospho/79.9663); N18(Deamidated/0.9840)	T,N,Y,N	435,437,439,445	187.07 - 187.07	HexNAc(1)Hex(2)
128	Y.SLPAINVTInnyNPssWNR.R	N10(Deamidated/0.9840); N11(Deamidated/0.9840); Y12(Phospho/79.9663); S15(OGlycan/656.2276); S16(OGlycan/568.2116)	N,N,Y,S,S	437,438,439,442,443	162.18 - 162.18	HexNAc(2)Hex(1),HexNAc(1)Hex(1)NeuAc(1)
129	Y.SLPAINVTINNYNPssW.N	S15(OGlycan/947.3230); S16(OGlycan/656.2276)	S,S	442,443	64.74 - 67.43	HexNAc(1)Hex(1)NeuAc(1), HexNAc(1)Hex(1)NeuAc(2)
130	Y.SLPAINVTINNYNPssW.N	S15(OGlycan/947.3230); S16(OGlycan/818.2804)	S,S	442,443	145.32 - 145.32	HexNAc(1)Hex(1)NeuAc(2), HexNAc(1)Hex(2)NeuAc(1)
131	F.NLssHSVVY.S	S3(OGlycan/1021.3598); S4(OGlycan/730.2644)	S,S	456,457	194.27 - 255.06	HexNAc(2)Hex(2),HexNAc(2)Hex(2)NeuAc(1)

132	F.NLssHSVVYSRy.C	S3(OGlycan/1062.3864); S4(OGlycan/730.2644); Y12(Phospho/79.9663)	S,S,Y	456,457,465	46.85 - 46.85	HexNAc(2)Hex(2),HexNAc(3)Hex(1)NeuAc(1)
133	F.NLssHSVVYSRy.C	S3(OGlycan/1167.4177); S4(OGlycan/1062.3864); Y12(Phospho/79.9663)	S,S,Y	456,457,465	138.86 - 138.86	HexNAc(3)Hex(1)NeuAc(1), HexNAc(2)Hex(2)Fuc(1)NeuAc(1)
134	F.NLssHSVVY.S	S3(OGlycan/365.1322); S4(OGlycan/527.1850)	S,S	456,457	195.90 - 317.38	HexNAc(1)Hex(1)HexNAc(1)Hex(2)
135	F.NLssHSVVY.S	S3(OGlycan/365.1322); S4(OGlycan/730.2644)	S,S	456,457	334.75 - 334.75	HexNAc(1)Hex(1)HexNAc(2)Hex(2)
136	F.NLsSHsVVY.S	S3(OGlycan/365.1322); S6(OGlycan/730.2644)	S,S	456,459	384.10 - 384.10	HexNAc(1)Hex(1)HexNAc(2)Hex(2)
137	F.NLssHSVVY.S	S3(OGlycan/527.1850); S4(OGlycan/730.2644)	S,S	456,457	144.80 - 144.80	HexNAc(2)Hex(2)HexNAc(1)Hex(2)
138	F.NLssHSVVY.S	S3(OGlycan/568.2116); S4(OGlycan/527.1850)	S,S	456,457	269.38 - 325.45	HexNAc(2)Hex(1)HexNAc(1)Hex(2)
139	F.NLssHSVVY.S	S3(OGlycan/568.2116); S4(OGlycan/730.2644)	S,S	456,457	275.59 - 308.88	HexNAc(2)Hex(1)HexNAc(2)Hex(2)
140	F.NLssHSVVY.S	S3(OGlycan/714.2695); S4(OGlycan/730.2644)	S,S	456,457	258.93 - 264.23	HexNAc(2)Hex(2),HexNAc(2)Hex(1)Fuc(1)
141	F.NLssHSVVY.S	S3(OGlycan/730.2644); S4(OGlycan/1021.3598)	S,S	456,457	154.46 - 338.81	HexNAc(2)Hex(2),HexNAc(2)Hex(2)NeuAc(1)
142	F.NLssHSVVYSRy.C	S3(OGlycan/730.2644); S4(OGlycan/1062.3864); Y12(Phospho/79.9663)	S,S,Y	456,457,465	169.58 - 169.58	HexNAc(2)Hex(2),HexNAc(3)Hex(1)NeuAc(1)
143	F.NLssHSVVY.S	S3(OGlycan/730.2644); S4(OGlycan/527.1850)	S,S	456,457	167.91 - 167.91	HexNAc(2)Hex(2),HexNAc(1)Hex(2)
144	F.NLssHSVVySR.Y	S3(OGlycan/730.2644); S4(OGlycan/527.1850); Y9(Phospho/79.9663)	S,S,Y	456,457,462	127.98 - 127.98	HexNAc(2)Hex(2),HexNAc(1)Hex(2)
145	F.NLssHSVVY.S	S3(OGlycan/730.2644); S4(OGlycan/714.2695)	S,S	456,457	296.48 - 296.48	HexNAc(2)Hex(2),HexNAc(2)Hex(1)Fuc(1)
146	F.NLssHSVVY.S	S3(OGlycan/730.2644); S4(OGlycan/818.2804)	S,S	456,457	238.62 - 238.62	HexNAc(2)Hex(2),HexNAc(1)Hex(2)NeuAc(1)
147	F.NLssHSVVY.S	S3(OGlycan/818.2804); S4(OGlycan/730.2644)	S,S	456,457	214.00 - 340.57	HexNAc(2)Hex(2),HexNAc(1)Hex(2)NeuAc(1)

148	F.NLssHSVVY.S	S3(OGlycan/527.1850); S4(OGlycan/527.1850)	S,S	456,457	303.52 - 395.97	HexNAc(1)Hex(2),HexNAc(1)Hex(2)
149	F.NLsSHSVVYSRy.C	S3(OGlycan/714.2695); Y12(Phospho/79.9663)	S,Y	456,465	139.72 - 139.72	HexNAc(2)Hex(1)Fuc(1)
150	F.NLsSHSVVYSRy.C	S3(OGlycan/730.2644); Y12(Phospho/79.9663)	S,Y	456,465	90.08 - 161.27	HexNAc(2)Hex(2)
151	F.NLssHSVVY.S	S3(OGlycan/527.1850); S4(OGlycan/1167.4177)	S,S	456,457	134.99 - 411.30	HexNAc(2)Hex(2)Fuc(1)NeuAc(1),HexNAc(1)Hex(2)
152	F.NLssHSVVYSRy.C	S3(OGlycan/568.2116); S4(OGlycan/714.2695); Y12(Phospho/79.9663)	S,S,Y	456,457,465	154.51 - 154.51	HexNAc(2)Hex(1),HexNAc(2)Hex(1)Fuc(1)
153	F.NLssHSVVY.S	S3(OGlycan/714.2695); S4(OGlycan/1167.4177)	S,S	456,457	387.70 - 415.31	HexNAc(2)Hex(2)Fuc(1)NeuAc(1),HexNAc(2)Hex(1)Fuc(1)
154	F.NLssHSVVY.S	S3(OGlycan/730.2644); S4(OGlycan/1167.4177)	S,S	456,457	161.66 - 225.72	HexNAc(2)Hex(2),HexNAc(2)Hex(2)Fuc(1)NeuAc(1)
155	F.NLssHSVVySR.Y	S3(OGlycan/730.2644); S4(OGlycan/1167.4177); Y9(Phospho/79.9663)	S,S,Y	456,457,462	59.99 - 59.99	HexNAc(2)Hex(2),HexNAc(2)Hex(2)Fuc(1)NeuAc(1)
156	F.NLssHSVVY.S	S3(OGlycan/818.2804); S4(OGlycan/1167.4177)	S,S	456,457	128.24 - 128.24	HexNAc(2)Hex(2)Fuc(1)NeuAc(1),HexNAc(1)Hex(2)NeuAc(1)
157	F.NLsSHSVVY.S	S3(OGlycan/730.2644)	S	456	100.89 - 100.89	HexNAc(2)Hex(2)
158	F.NLsSHSVVY.S	S3(OGlycan/568.2116)	S	456	338.06 - 450.02	HexNAc(2)Hex(1)
159	F.NLssHSVVY.S	S3(OGlycan/365.1322); S4(OGlycan/365.1322)	S,S	456,457	164.62 - 164.62	HexNAc(1)Hex(1),HexNAc(1)Hex(1)
160	F.NLsSHsVVY.S	S3(OGlycan/947.3230); S6(OGlycan/1062.3864)	S,S	456,459	350.73 - 350.73	HexNAc(1)Hex(1)NeuAc(2), HexNAc(3)Hex(1)NeuAc(1)
161	F.NLssHSVVY.S	S3(OGlycan/714.2695); S4(OGlycan/714.2695)	S,S	456,457	252.95 - 254.87	HexNAc(2)Hex(1)Fuc(1)HexNAc(2)Hex(1)Fuc(1)
162	F.NLssHSVVY.S	S3(OGlycan/730.2644); S4(OGlycan/730.2644)	S,S	456,457	208.65 - 387.73	HexNAc(2)Hex(2),HexNAc(2)Hex(2)
163	F.NLssHSVVY.S	S3(OGlycan/947.3230); S4(OGlycan/947.3230)	S,S	456,457	212.91 - 339.99	HexNAc(1)Hex(1)NeuAc(2), HexNAc(1)Hex(1)NeuAc(2)

164	F.NNFNLssHSVVY.S	S6(OGlycan/527.1850); S7(OGlycan/527.1850)	S,S	456,457	73.33 - 73.33	HexNAc(1)Hex(2),HexNAc(1)Hex(2)
165	F.NLSsHsVVYSRy.C	S4(OGlycan/1062.3864); S6(OGlycan/1167.4177); Y12(Phospho/79.9663)	S,S,Y	457,459,465	165.96 - 194.10	HexNAc(3)Hex(1)NeuAc(1), HexNAc(2)Hex(2)Fuc(1)NeuAc(1)
166	F.NLSsHsVVYSRy.C	S4(OGlycan/365.1322); S6(OGlycan/511.1901); Y12(Phospho/79.9663)	S,S,Y	457,459,465	152.62 - 152.62	HexNAc(1)Hex(1),HexNAc(1)Hex(1)Fuc(1)
167	F.NLSsHsVVYsR.Y	S4(OGlycan/365.1322); S6(OGlycan/568.2116); S10(Phospho/79.9663)	S,S,S	457,459,463	168.09 - 168.09	HexNAc(1)Hex(1),HexNAc(2)Hex(1)
168	F.NLSsHsVVYSRy.C	S4(OGlycan/365.1322); S6(OGlycan/568.2116); Y12(Phospho/79.9663)	S,S,Y	457,459,465	153.08 - 153.08	HexNAc(1)Hex(1),HexNAc(2)Hex(1)
169	F.NLSsHsVVYSRy.C	S4(OGlycan/511.1901); S6(OGlycan/730.2644); Y12(Phospho/79.9663)	S,S,Y	457,459,465	195.12 - 195.12	HexNAc(1)Hex(1)Fuc(1),HexNAc(2)Hex(2)
170	F.NLSsHsVVY.S	S4(OGlycan/527.1850); S6(OGlycan/365.1322)	S,S	457,459	508.58 - 523.98	HexNAc(1)Hex(1),HexNAc(1)Hex(2)
171	F.NLSsHsVVY.S	S4(OGlycan/714.2695); S6(OGlycan/730.2644)	S,S	457,459	208.51 - 208.51	HexNAc(2)Hex(2),HexNAc(2)Hex(1)Fuc(1)
172	F.NLSsHsVVY.S	S4(OGlycan/730.2644); S6(OGlycan/527.1850)	S,S	457,459	241.17 - 382.31	HexNAc(2)Hex(2),HexNAc(1)Hex(2)
173	F.NLSsHsVVY.S	S4(OGlycan/527.1850); S6(OGlycan/527.1850)	S,S	457,459	353.79 - 557.72	HexNAc(1)Hex(2),HexNAc(1)Hex(2)
174	F.NLSsHsVVYSRy.C	S4(OGlycan/714.2695); S6(OGlycan/1062.3864); Y12(Phospho/79.9663)	S,S,Y	457,459,465	144.67 - 154.35	HexNAc(3)Hex(1)NeuAc(1), HexNAc(2)Hex(1)Fuc(1)
175	F.NLSsHsVVYSRy.C	S4(OGlycan/714.2695); S6(OGlycan/714.2695); Y12(Phospho/79.9663)	S,S,Y	457,459,465	142.77 - 142.77	HexNAc(2)Hex(1)Fuc(1),HexNAc(2)Hex(1)Fuc(1)
176	F.NLSsHsVVYSRy.C	S4(OGlycan/730.2644); S6(OGlycan/730.2644); Y12(Phospho/79.9663)	S,S,Y	457,459,465	156.85 - 163.05	HexNAc(2)Hex(2),HexNAc(2)Hex(2)
177	F.NLSsHsVVYSRy.C	S4(OGlycan/527.1850); S6(OGlycan/527.1850); Y12(Phospho/79.9663)	S,S,Y	457,459,465	87.54 - 87.54	HexNAc(1)Hex(2),HexNAc(1)Hex(2)

178	F.NLSsHsVVYSRy.C	S4(OGlycan/730.2644); S6(OGlycan/527.1850); Y12(Phospho/79.9663)	S,S,Y	457,459,465	163.18 - 163.18	HexNAc(2)Hex(2),HexNAc(1)Hex(2)
179	F.NLSsHsVVY.S	S4(OGlycan/730.2644); S6(OGlycan/568.2116)	S,S	457,459	311.54 - 311.54	HexNAc(2)Hex(1),HexNAc(2)Hex(2)
180	F.NLSsHsVVY.S	S4(OGlycan/730.2644); S6(OGlycan/730.2644)	S,S	457,459	289.78 - 388.70	HexNAc(2)Hex(2),HexNAc(2)Hex(2)
181	F.NLSsHsVVY.S	S4(OGlycan/365.1322); S6(OGlycan/527.1850)	S,S	457,459	548.97 - 548.97	HexNAc(1)Hex(1),HexNAc(1)Hex(2)
182	F.NLSSHsVVYSRy.C	S6(OGlycan/730.2644); Y12(Phospho/79.9663)	S,Y	459,465	135.61 - 135.61	HexNAc(2)Hex(2)
183	F.NLSSHsVVY.S	S6(OGlycan/730.2644)	S	459	170.48 - 170.48	HexNAc(2)Hex(2)
184	K.sHKPPSASCPIGTNY.R	S1(OGlycan/1167.4177)	S	487	220.83 - 261.89	HexNAc(2)Hex(2)Fuc(1)NeuAc(1)
185	K.sHKPPSASCPIGTNYR.S	S1(OGlycan/1312.4552)	S	487	371.41 - 371.41	HexNAc(2)Hex(2)NeuAc(2)
186	K.sHKPPSASCPIGTNY.R	S1(OGlycan/1312.4552)	S	487	169.12 - 302.19	HexNAc(2)Hex(2)NeuAc(2)
187	K.sHKPPSASCPIGTNY.R	S1(OGlycan/656.2276)	S	487	216.85 - 368.65	HexNAc(1)Hex(1)NeuAc(1)
188	K.sHKPPSASCPIGTNY.R	S1(OGlycan/494.1748)	S	487	297.71 - 297.71	HexNAc(1)NeuAc(1)
189	K.SHKPPsASCPIGTNYR.S	S6(OGlycan/1312.4552)	S	492	330.51 - 382.69	HexNAc(2)Hex(2)NeuAc(2)
190	K.SHKPPsASCPIGTNY.R	S6(OGlycan/1312.4552)	S	492	202.92 - 202.92	HexNAc(2)Hex(2)NeuAc(2)
191	K.PPsAsCPIGTNYR.S	S3(OGlycan/527.1850); S5(OGlycan/730.2644)	S,S	492,494	186.31 - 186.31	HexNAc(2)Hex(2),HexNAc(1)Hex(2)
192	K.PPsAsCPIGTNYR.S	S3(OGlycan/527.1850); S5(OGlycan/818.2804)	S,S	492,494	76.76 - 76.76	HexNAc(1)Hex(2),HexNAc(1)Hex(2)NeuAc(1)
193	K.PPsAsCPIGTNYR.S	S3(OGlycan/730.2644); S5(OGlycan/365.1322)	S,S	492,494	65.02 - 65.02	HexNAc(1)Hex(1),HexNAc(2)Hex(2)
194	K.PPsAsCPIGTNYR.S	S3(OGlycan/818.2804); S5(OGlycan/818.2804)	S,S	492,494	64.48 - 65.72	HexNAc(1)Hex(2)NeuAc(1), HexNAc(1)Hex(2)NeuAc(1)

195	K.SHKPPSAsCPIGTNY.R	S8(OGlycan/656.2276)	S	494	404.72 - 404.72	HexNAc(1)Hex(1)NeuAc(1)
196	K.SHKPPSAsCPIGTNY.R	S8(OGlycan/947.3230)	S	494	231.71 - 782.97	HexNAc(1)Hex(1)NeuAc(2)
197	K.SHKPPSASCPIGtNY.R	T13(OGlycan/656.2276)	T	499	402.14 - 402.14	HexNAc(1)Hex(1)NeuAc(1)
198	K.SHKPPSASCPIGtNY.R	T13(OGlycan/494.1748)	T	499	225.90 - 332.35	HexNAc(1)NeuAc(1)
199	R.sCEstTVLDHTDW.C	S1(Phospho/79.9663); S4(OGlycan/494.1748); T5(OGlycan/527.1850)	S,S,T	503,506,507	82.65 - 82.65	HexNAc(1)Hex(2),HexNAc(1)NeuAc(1)
200	R.sCEsTTVLDHTDW.C	S1(Phospho/79.9663); S4(OGlycan/568.2116)	S,S	503,506	33.85 - 76.80	HexNAc(2)Hex(1)
201	R.sCEsTTVLDHTDW.C	S1(Phospho/79.9663); S4(OGlycan/656.2276)	S,S	503,506	101.26 - 101.26	HexNAc(1)Hex(1)NeuAc(1)
202	Y.RsCEsTTVLDHTDW.C	S2(OGlycan/947.3230); S5(OGlycan/947.3230)	S,S	503,506	97.28 - 150.61	HexNAc(1)Hex(1)NeuAc(2), HexNAc(1)Hex(1)NeuAc(2)
203	R.SCEstTVLDHTDW.C	S4(OGlycan/947.3230); T5(OGlycan/947.3230)	S,T	506,507	156.93 - 267.28	HexNAc(1)Hex(1)NeuAc(2), HexNAc(1)Hex(1)NeuAc(2)
204	R.SCEStTVLDHTDW.C	T5(OGlycan/1312.4552)	T	507	69.50 - 69.50	HexNAc(2)Hex(2)NeuAc(2)
205	R.CSCLPDPItAYDPR.S	T9(OGlycan/1312.4552)	T	526	405.14 - 405.14	HexNAc(2)Hex(2)NeuAc(2)
206	R.sCsQK.K	S1(OGlycan/1167.4177); S3(OGlycan/1062.3864)	S,S	532,534	66.47 - 66.47	HexNAc(3)Hex(1)NeuAc(1), HexNAc(2)Hex(2)Fuc(1)NeuAc(1)
207	R.sCsQK.K	S1(OGlycan/1167.4177); S3(OGlycan/1312.4552)	S,S	532,534	65.89 - 65.89	HexNAc(2)Hex(2)Fuc(1)NeuAc(1),HexNAc(2)Hex(2)NeuAc(2)
208	R.sCsQK.K	S1(OGlycan/1167.4177); S3(OGlycan/1167.4177)	S,S	532,534	55.98 - 55.98	HexNAc(2)Hex(2)Fuc(1)NeuAc(1),HexNAc(2)Hex(2)Fuc(1)NeuAc(1)
209	K.KsLVGVGEHCAGFGVDEEK.C	S2(OGlycan/947.3230)	S	538	73.50 - 73.50	HexNAc(1)Hex(1)NeuAc(2)
210	F.GVDEEKCGVLDGsY.N	S13(OGlycan/818.2804)	S	562	56.45 - 70.25	HexNAc(1)Hex(2)NeuAc(1)

211	F.GVDEEKCGVLDGsY.N	S13(OGlycan/1021.3598)	S	562	111.55 - 180.21	HexNAc(2)Hex(2)NeuAc(1)
212	F.GVDEEKCGVLDGsY.N	S13(OGlycan/656.2276)	S	562	79.54 - 79.54	HexNAc(1)Hex(1)NeuAc(1)
213	W.sYDTCVsNNR.C	S1(OGlycan/365.1322); S7(OGlycan/527.1850)	S,S	578,584	453.99 - 453.99	HexNAc(1)Hex(1),HexNAc(1)Hex(2)
214	W.sYDtCVSNNR.C	S1(OGlycan/365.1322); T4(OGlycan/714.2695)	S,T	578,581	311.74 - 311.74	HexNAc(1)Hex(1),HexNAc(2)Hex(1)Fuc(1)
215	W.sYDtCVSNNR.C	S1(OGlycan/730.2644); T4(OGlycan/714.2695)	S,T	578,581	130.01 - 227.81	HexNAc(2)Hex(2),HexNAc(2)Hex(1)Fuc(1)
216	W.sYDTCVsNNR.C	S1(OGlycan/947.3230); S7(OGlycan/656.2276)	S,S	578,584	105.22 - 105.22	HexNAc(1)Hex(1)NeuAc(1), HexNAc(1)Hex(1)NeuAc(2)
217	W.sYDtCVSnNR.C	S1(OGlycan/730.2644); T4(OGlycan/714.2695); N8(Deamidated/0.9840)	S,T,N	578,581,585	184.79 - 184.79	HexNAc(2)Hex(2),HexNAc(2)Hex(1)Fuc(1)
218	W.sYDtCVSNNR.C	S1(OGlycan/527.1850); T4(OGlycan/714.2695)	S,T	578,581	173.26 - 276.81	HexNAc(2)Hex(1)Fuc(1),HexNAc(1)Hex(2)
219	W.sYDtCVSNNR.C	S1(OGlycan/730.2644); T4(OGlycan/1021.3598)	S,T	578,581	195.36 - 195.36	HexNAc(2)Hex(2),HexNAc(2)Hex(2)NeuAc(1)
220	W.sYDtCVSNNR.C	S1(OGlycan/730.2644); T4(OGlycan/1167.4177)	S,T	578,581	125.92 - 199.13	HexNAc(2)Hex(2),HexNAc(2)Hex(2)Fuc(1)NeuAc(1)
221	W.sYDtCVSNNR.C	S1(OGlycan/494.1748); T4(OGlycan/947.3230)	S,T	578,581	82.84 - 82.84	HexNAc(1)Hex(1)NeuAc(2), HexNAc(1)NeuAc(1)
222	W.sYDtCVSNNR.C	S1(OGlycan/527.1850); T4(OGlycan/730.2644)	S,T	578,581	209.98 - 209.98	HexNAc(2)Hex(2),HexNAc(1)Hex(2)
223	W.sYDTCVsNNR.C	S1(OGlycan/730.2644); S7(OGlycan/714.2695)	S,S	578,584	248.79 - 317.69	HexNAc(2)Hex(2),HexNAc(2)Hex(1)Fuc(1)
224	W.sYDTCVsNNR.C	S1(OGlycan/568.2116); S7(OGlycan/714.2695)	S,S	578,584	163.42 - 213.15	HexNAc(2)Hex(1)HexNAc(2)Hex(1)Fuc(1)
225	W.sYDtCVSNNR.C	S1(OGlycan/527.1850); T4(OGlycan/568.2116)	S,T	578,581	48.43 - 48.43	HexNAc(2)Hex(1),HexNAc(1)Hex(2)
226	W.sYDTCVsNNR.C	S1(OGlycan/527.1850); S7(OGlycan/714.2695)	S,S	578,584	258.01 - 309.10	HexNAc(2)Hex(1)Fuc(1),HexNAc(1)Hex(2)
227	W.sYDTCVsNNR.C	S1(OGlycan/365.1322); S7(OGlycan/714.2695)	S,S	578,584	343.09 - 356.94	HexNAc(1)Hex(1),HexNAc(2)Hex(1)Fuc(1)

228	W.sYDtCVSnNR.C	S1(OGlycan/730.2644); T4(OGlycan/568.2116); N8(Deamidated/0.9840)	S,T,N	578,581,585	164.24 - 164.24	HexNAc(2)Hex(1),HexNAc(2)Hex(2)
229	W.sYDtCVSNNR.C	S1(OGlycan/568.2116); T4(OGlycan/714.2695)	S,T	578,581	203.77 - 203.77	HexNAc(2)Hex(1),HexNAc(2)Hex(1)Fuc(1)
230	W.SYDtCVsNNR.C	T4(OGlycan/1167.4177); S7(OGlycan/730.2644)	T,S	581,584	222.49 - 222.49	HexNAc(2)Hex(2),HexNAc(2)Hex(2)Fuc(1)NeuAc(1)
231	W.SYDtCVsNNR.C	T4(OGlycan/365.1322); S7(OGlycan/511.1901)	T,S	581,584	227.66 - 348.90	HexNAc(1)Hex(1),HexNAc(1)Hex(1)Fuc(1)
232	W.SYDtCVsNNR.C	T4(OGlycan/365.1322); S7(OGlycan/527.1850)	T,S	581,584	177.86 - 424.53	HexNAc(1)Hex(1),HexNAc(1)Hex(2)
233	W.SYDtCVsNNR.C	T4(OGlycan/365.1322); S7(OGlycan/730.2644)	T,S	581,584	250.80 - 384.66	HexNAc(1)Hex(1),HexNAc(2)Hex(2)
234	W.SYDtCVsNNR.C	T4(OGlycan/511.1901); S7(OGlycan/365.1322)	T,S	581,584	308.13 - 308.13	HexNAc(1)Hex(1),HexNAc(1)Hex(1)Fuc(1)
235	W.SYDtCVsNNR.C	T4(OGlycan/511.1901); S7(OGlycan/527.1850)	T,S	581,584	313.25 - 313.25	HexNAc(1)Hex(1)Fuc(1),HexNAc(1)Hex(2)
236	W.SYDtCVsNNR.C	T4(OGlycan/527.1850); S7(OGlycan/365.1322)	T,S	581,584	241.16 - 560.72	HexNAc(1)Hex(1),HexNAc(1)Hex(2)
237	W.SYDtCVsNNR.C	T4(OGlycan/656.2276); S7(OGlycan/947.3230)	T,S	581,584	276.72 - 276.72	HexNAc(1)Hex(1)NeuAc(1), HexNAc(1)Hex(1)NeuAc(2)
238	W.SYDtCVsNNR.C	T4(OGlycan/730.2644); S7(OGlycan/1021.3598)	T,S	581,584	248.13 - 248.13	HexNAc(2)Hex(2),HexNAc(2)Hex(2)NeuAc(1)
239	W.SYDtCVsNNR.C	T4(OGlycan/730.2644); S7(OGlycan/365.1322)	T,S	581,584	269.44 - 269.44	HexNAc(1)Hex(1),HexNAc(2)Hex(2)
240	W.SYDtCVsNNR.C	T4(OGlycan/730.2644); S7(OGlycan/511.1901)	T,S	581,584	167.34 - 167.34	HexNAc(1)Hex(1)Fuc(1),HexNAc(2)Hex(2)
241	W.SYDtCVsNNR.C	T4(OGlycan/730.2644); S7(OGlycan/527.1850)	T,S	581,584	228.26 - 228.26	HexNAc(2)Hex(2),HexNAc(1)Hex(2)
242	W.SYDtCVsNNR.C	T4(OGlycan/527.1850); S7(OGlycan/511.1901)	T,S	581,584	239.10 - 319.98	HexNAc(1)Hex(1)Fuc(1),HexNAc(1)Hex(2)
243	W.SYDtCVsNNR.C	T4(OGlycan/527.1850); S7(OGlycan/568.2116)	T,S	581,584	340.20 - 458.92	HexNAc(2)Hex(1),HexNAc(1)Hex(2)
244	W.SYDtCVsNNR.C	T4(OGlycan/527.1850); S7(OGlycan/527.1850)	T,S	581,584	400.12 - 534.34	HexNAc(1)Hex(2),HexNAc(1)Hex(2)

245	W.SYDtCVsNNR.C	T4(OGlycan/730.2644); S7(OGlycan/730.2644)	T,S	581,584	204.63 - 448.89	HexNAc(2)Hex(2),HexNAc(2)Hex(2)
246	W.SYDtCVsNNR.C	T4(OGlycan/527.1850); S7(OGlycan/730.2644)	T,S	581,584	461.30 - 461.30	HexNAc(2)Hex(2),HexNAc(1)Hex(2)
247	W.SYDTCVsNNR.C	S7(OGlycan/730.2644)	S	584	433.48 - 563.04	HexNAc(2)Hex(2)
248	W.SYDTCVsNNR.C	S7(OGlycan/568.2116)	S	584	498.98 - 539.01	HexNAc(2)Hex(1)
249	F.KDFVtNK.T	T5(OGlycan/730.2644)	T	665	348.47 - 453.35	HexNAc(2)Hex(2)
250	F.KDFVtNK.T	T5(OGlycan/568.2116)	T	665	546.80 - 575.08	HexNAc(2)Hex(1)
251	Y.AGRVsAAF.H	S5(OGlycan/1021.3598)	S	680	212.82 - 224.66	HexNAc(2)Hex(2)NeuAc(1)
252	Y.AGRVsAAF.H	S5(OGlycan/1312.4552)	S	680	228.97 - 260.76	HexNAc(2)Hex(2)NeuAc(2)
253	R.VsAAF.H	S2(OGlycan/1312.4552)	S	680	372.40 - 390.30	HexNAc(2)Hex(2)NeuAc(2)
254	R.VsAAF.H	S2(OGlycan/947.3230)	S	680	325.84 - 424.74	HexNAc(1)Hex(1)NeuAc(2)
255	R.VsAAF.H	S2(OGlycan/656.2276)	S	680	379.78 - 379.78	HexNAc(1)Hex(1)NeuAc(1)
256	Y.VLnnISLTtQPY.F	N3(Deamidated/0.9840); N4(Deamidated/0.9840); T9(OGlycan/1312.4552)	N,N,T	704,705,710	208.00 - 380.87	HexNAc(2)Hex(2)NeuAc(2)
257	Y.VLnNIsLTTQPY.F	N3(Deamidated/0.9840); S6(OGlycan/1021.3598)	N,S	704,707	236.69 - 295.62	HexNAc(2)Hex(2)NeuAc(1)
258	Y.VLnNIsLTTQPYFDSY.L	N3(Deamidated/0.9840); S6(OGlycan/1021.3598)	N,S	704,707	149.33 - 353.05	HexNAc(2)Hex(2)NeuAc(1)
259	Y.VLnNIsLTTQPY.F	N3(Deamidated/0.9840); S6(OGlycan/1062.3864)	N,S	704,707	205.99 - 350.68	HexNAc(3)Hex(1)NeuAc(1)
260	Y.VLnNIsLTTQPYFDSY.L	N3(Deamidated/0.9840); S6(OGlycan/1062.3864)	N,S	704,707	150.81 - 232.66	HexNAc(3)Hex(1)NeuAc(1)
261	Y.VLnNIsLTTQPy.F	N3(Deamidated/0.9840); S6(OGlycan/1167.4177);	N,S,Y	704,707,713	115.95 - 115.95	HexNAc(2)Hex(2)Fuc(1)NeuAc(1)

		Y12(Phospho/79.9663)				
262	Y.VLnNIsLTTQPYFDSY.L	N3(Deamidated/0.9840); S6(OGlycan/1312.4552)	N,S	704,707	215.33 - 215.33	HexNAc(2)Hex(2)NeuAc(2)
263	Y.VLnNIsLTTQPYF.D	N3(Deamidated/0.9840); S6(OGlycan/1312.4552)	N,S	704,707	91.16 - 91.16	HexNAc(2)Hex(2)NeuAc(2)
264	Y.VLnNIsLTTQPY.F	N3(Deamidated/0.9840); S6(OGlycan/365.1322)	N,S	704,707	60.17 - 60.17	HexNAc(1)Hex(1)
265	Y.VLnNIsLtTQPYFDSY.L	N3(Deamidated/0.9840); S6(OGlycan/365.1322); T8(OGlycan/1021.3598)	N,S,T	704,707,709	46.38 - 46.38	HexNAc(1)Hex(1),HexNAc(2)Hex(2)NeuAc(1)
266	Y.VLnNIsLtTQPYFDSY.L	N3(Deamidated/0.9840); S6(OGlycan/365.1322); T8(OGlycan/1167.4177)	N,S,T	704,707,709	71.66 - 102.52	HexNAc(1)Hex(1),HexNAc(2)Hex(2)Fuc(1)NeuAc(1)
267	Y.VLnNIsLtTQPY.F	N3(Deamidated/0.9840); S6(OGlycan/511.1901); T8(OGlycan/818.2804)	N,S,T	704,707,709	183.91 - 183.91	HexNAc(1)Hex(1)Fuc(1),HexNAc(1)Hex(2)NeuAc(1)
268	Y.VLnNIsLTTQPY.F	N3(Deamidated/0.9840); S6(OGlycan/568.2116)	N,S	704,707	245.11 - 270.62	HexNAc(2)Hex(1)
269	Y.VLnNIsLTTQPYFDSY.L	N3(Deamidated/0.9840); S6(OGlycan/656.2276)	N,S	704,707	314.97 - 314.97	HexNAc(1)Hex(1)NeuAc(1)
270	Y.VLnNIsLTTQPY.F	N3(Deamidated/0.9840); S6(OGlycan/656.2276)	N,S	704,707	193.45 - 400.76	HexNAc(1)Hex(1)NeuAc(1)
271	Y.VLnNIsLTTQPY.F	N3(Deamidated/0.9840); S6(OGlycan/947.3230)	N,S	704,707	178.87 - 178.87	HexNAc(1)Hex(1)NeuAc(2)
272	Y.VLnNIsLTTQPYFDSY.L	N3(Deamidated/0.9840); S6(OGlycan/947.3230)	N,S	704,707	94.57 - 94.57	HexNAc(1)Hex(1)NeuAc(2)
273	Y.VLnNIsLtTQPY.F	N3(Deamidated/0.9840); S6(OGlycan/947.3230); T8(OGlycan/511.1901)	N,S,T	704,707,709	218.76 - 218.76	HexNAc(1)Hex(1)Fuc(1),HexNAc(1)Hex(1)NeuAc(2)
274	Y.VLnNISLtTQPY.F	N3(Deamidated/0.9840); T8(OGlycan/1021.3598)	N,T	704,709	236.42 - 429.51	HexNAc(2)Hex(2)NeuAc(1)
275	Y.VLnNISLttQPY.F	N3(Deamidated/0.9840); T8(OGlycan/1021.3598); T9(OGlycan/656.2276)	N,T,T	704,709,710	377.14 - 482.33	HexNAc(1)Hex(1)NeuAc(1), HexNAc(2)Hex(2)NeuAc(1)

276	Y.VLnNISLtTQPY.F	N3(Deamidated/0.9840); T8(OGlycan/1062.3864)	N,T	704,709	303.51 - 436.42	HexNAc(3)Hex(1)NeuAc(1)
277	Y.VLnNISLtTQPy.F	N3(Deamidated/0.9840); T8(OGlycan/1062.3864); Y12(Phospho/79.9663)	N,T,Y	704,709,713	165.64 - 358.61	HexNAc(3)Hex(1)NeuAc(1)
278	Y.VLnNISLtTQPY.F	N3(Deamidated/0.9840); T8(OGlycan/1167.4177)	N,T	704,709	313.49 - 448.01	HexNAc(2)Hex(2)Fuc(1)NeuAc(1)
279	Y.VLnNISLttQPY.F	N3(Deamidated/0.9840); T8(OGlycan/1167.4177); T9(OGlycan/656.2276)	N,T,T	704,709,710	238.56 - 238.56	HexNAc(1)Hex(1)NeuAc(1), HexNAc(2)Hex(2)Fuc(1)NeuAc(1)
280	Y.VLnNISLttQPY.F	N3(Deamidated/0.9840); T8(OGlycan/1312.4552); T9(OGlycan/365.1322)	N,T,T	704,709,710	393.42 - 393.42	HexNAc(1)Hex(1),HexNAc(2)Hex(2)NeuAc(2)
281	Y.VLnNISLtTQPY.F	N3(Deamidated/0.9840); T8(OGlycan/365.1322)	N,T	704,709	150.48 - 150.48	HexNAc(1)Hex(1)
282	Y.VLnNISLtTQPY.F	N3(Deamidated/0.9840); T8(OGlycan/494.1748)	N,T	704,709	352.30 - 352.30	HexNAc(1)NeuAc(1)
283	Y.VLnNISLttQPY.F	N3(Deamidated/0.9840); T8(OGlycan/494.1748); T9(OGlycan/714.2695)	N,T,T	704,709,710	385.67 - 385.67	HexNAc(2)Hex(1)Fuc(1),HexNAc(1)NeuAc(1)
284	Y.VLnNISLtTQPY.F	N3(Deamidated/0.9840); T8(OGlycan/656.2276)	N,T	704,709	258.23 - 631.86	HexNAc(1)Hex(1)NeuAc(1)
285	Y.VLnNISLttQPY.F	N3(Deamidated/0.9840); T8(OGlycan/714.2695); T9(OGlycan/494.1748)	N,T,T	704,709,710	365.99 - 392.66	HexNAc(2)Hex(1)Fuc(1),HexNAc(1)NeuAc(1)
286	Y.VLnNISLtTQPY.F	N3(Deamidated/0.9840); T8(OGlycan/730.2644)	N,T	704,709	351.72 - 351.72	HexNAc(2)Hex(2)
287	Y.VLnNISLttQPYF.D	N3(Deamidated/0.9840); T8(OGlycan/818.2804); T9(OGlycan/511.1901)	N,T,T	704,709,710	300.01 - 300.01	HexNAc(1)Hex(1)Fuc(1),HexNAc(1)Hex(2)NeuAc(1)
288	Y.VLnNISLTtQPYFDSY.L	N3(Deamidated/0.9840); T9(OGlycan/1312.4552)	N,T	704,710	99.38 - 99.38	HexNAc(2)Hex(2)NeuAc(2)
289	Y.VLnNISLTtQPy.F	N3(Deamidated/0.9840); T9(OGlycan/1312.4552); Y12(Phospho/79.9663)	N,T,Y	704,710,713	265.31 - 265.31	HexNAc(2)Hex(2)NeuAc(2)

290	Y.VLnNISLTtQPYFDsY.L	N3(Deamidated/0.9840); T9(OGlycan/730.2644); S15(OGlycan/656.2276)	N,T,S	704,710,716	76.10 - 76.10	HexNAc(1)Hex(1)NeuAc(1), HexNAc(2)Hex(2)
291	Y.VLnNISLTtQPYFDSY.L	N3(Deamidated/0.9840); T9(OGlycan/947.3230)	N,T	704,710	132.72 - 132.72	HexNAc(1)Hex(1)NeuAc(2)
292	Y.VLnNISLtTQPY.F	N3(Deamidated/0.9840); T8(OGlycan/947.3230)	N,T	704,709	778.01 - 778.01	HexNAc(1)Hex(1)NeuAc(2)
293	Y.VLnnIsLTTQPYFDSY.L	N3(Deamidated/0.9840); N4(Deamidated/0.9840); S6(OGlycan/1312.4552)	N,N,S	704,705,707	127.78 - 127.78	HexNAc(2)Hex(2)NeuAc(2)
294	Y.VLnnIsLTTQPY.F	N3(Deamidated/0.9840); N4(Deamidated/0.9840); S6(OGlycan/1312.4552)	N,N,S	704,705,707	99.50 - 99.50	HexNAc(2)Hex(2)NeuAc(2)
295	Y.VLnnIsLTTQPY.F	N3(Deamidated/0.9840); N4(Deamidated/0.9840); S6(OGlycan/859.3070)	N,N,S	704,705,707	195.67 - 195.67	HexNAc(2)Hex(1)NeuAc(1)
296	Y.VLNnIsLTTQPY.F	N4(Deamidated/0.9840); S6(OGlycan/1021.3598)	N,S	705,707	164.19 - 369.30	HexNAc(2)Hex(2)NeuAc(1)
297	Y.VLNnIsLTTQPY.F	N4(Deamidated/0.9840); S6(OGlycan/1062.3864)	N,S	705,707	207.66 - 418.76	HexNAc(3)Hex(1)NeuAc(1)
298	Y.VLNnIsLTTQPY.F	N4(Deamidated/0.9840); S6(OGlycan/1167.4177)	N,S	705,707	317.52 - 317.52	HexNAc(2)Hex(2)Fuc(1)NeuAc(1)
299	Y.VLNnIsLTTQPYFDSY.L	N4(Deamidated/0.9840); S6(OGlycan/1312.4552)	N,S	705,707	57.74 - 57.74	HexNAc(2)Hex(2)NeuAc(2)
300	Y.VLNnIsLtTQPY.F	N4(Deamidated/0.9840); S6(OGlycan/494.1748); T8(OGlycan/714.2695)	N,S,T	705,707,709	236.77 - 236.77	HexNAc(2)Hex(1)Fuc(1),HexNAc(1)NeuAc(1)
301	Y.VLNnIsLtTQPYFDSY.L	N4(Deamidated/0.9840); S6(OGlycan/511.1901); T8(OGlycan/947.3230)	N,S,T	705,707,709	128.52 - 198.79	HexNAc(1)Hex(1)Fuc(1),HexNAc(1)Hex(1)NeuAc(2)
302	Y.VLNnIsLTTQPYFDSY.L	N4(Deamidated/0.9840); S6(OGlycan/656.2276)	N,S	705,707	153.42 - 153.42	HexNAc(1)Hex(1)NeuAc(1)
303	Y.VLNnIsLTTQPY.F	N4(Deamidated/0.9840); S6(OGlycan/859.3070)	N,S	705,707	151.09 - 184.41	HexNAc(2)Hex(1)NeuAc(1)
304	Y.VLNnISLtTQPY.F	N4(Deamidated/0.9840); T8(OGlycan/1062.3864)	N,T	705,709	195.82 - 311.68	HexNAc(3)Hex(1)NeuAc(1)

305	Y.VLNnISLtTQPY.F	N4(Deamidated/0.9840); T8(OGlycan/1312.4552)	N,T	705,709	624.27 - 624.27	HexNAc(2)Hex(2)NeuAc(2)
306	Y.VLNnISLttQPY.F	N4(Deamidated/0.9840); T8(OGlycan/511.1901); T9(OGlycan/818.2804)	N,T,T	705,709,710	471.68 - 471.68	HexNAc(1)Hex(1)Fuc(1),HexNAc(1)Hex(2)NeuAc(1)
307	Y.VLNnISLttQPYFDSY.L	N4(Deamidated/0.9840); T8(OGlycan/511.1901); T9(OGlycan/818.2804)	N,T,T	705,709,710	393.51 - 393.51	HexNAc(1)Hex(1)Fuc(1),HexNAc(1)Hex(2)NeuAc(1)
308	Y.VLNnISLttQPYF.D	N4(Deamidated/0.9840); T8(OGlycan/511.1901); T9(OGlycan/818.2804)	N,T,T	705,709,710	163.26 - 163.26	HexNAc(1)Hex(1)Fuc(1),HexNAc(1)Hex(2)NeuAc(1)
309	Y.VLNnISLtTQPY.F	N4(Deamidated/0.9840); T8(OGlycan/656.2276)	N,T	705,709	645.20 - 684.67	HexNAc(1)Hex(1)NeuAc(1)
310	Y.VLNnISLTtQPYFDSY.L	N4(Deamidated/0.9840); T9(OGlycan/656.2276)	N,T	705,710	376.57 - 436.33	HexNAc(1)Hex(1)NeuAc(1)
311	Y.VLNnISLTtQPY.F	N4(Deamidated/0.9840); T9(OGlycan/1312.4552)	N,T	705,710	650.88 - 682.18	HexNAc(2)Hex(2)NeuAc(2)
312	Y.VLNnISLtTQPY.F	N4(Deamidated/0.9840); T8(OGlycan/1021.3598)	N,T	705,709	173.71 - 671.15	HexNAc(2)Hex(2)NeuAc(1)
313	Y.VLNnISLtTQPY.F	N4(Deamidated/0.9840); T8(OGlycan/947.3230)	N,T	705,709	290.95 - 743.83	HexNAc(1)Hex(1)NeuAc(2)
314	Y.VLNnISLTtQPY.F	N4(Deamidated/0.9840); T9(OGlycan/656.2276)	N,T	705,710	651.07 - 651.07	HexNAc(1)Hex(1)NeuAc(1)
315	Y.VLNnISLTtQPY.F	N4(Deamidated/0.9840); T9(OGlycan/947.3230)	N,T	705,710	710.32 - 710.32	HexNAc(1)Hex(1)NeuAc(2)
316	Y.VLNnIsLTTQPYFDSY.L	N4(Deamidated/0.9840); S6(OGlycan/1062.3864)	N,S	705,707	186.57 - 186.57	HexNAc(3)Hex(1)NeuAc(1)
317	Y.VLNnIsLTTQPY.F	N4(Deamidated/0.9840); S6(OGlycan/494.1748)	N,S	705,707	201.92 - 201.92	HexNAc(1)NeuAc(1)
318	Y.VLNNIsLtTQPYF.D	S6(OGlycan/511.1901); T8(OGlycan/818.2804)	S,T	707,709	169.31 - 169.31	HexNAc(1)Hex(1)Fuc(1),HexNAc(1)Hex(2)NeuAc(1)
319	Y.VLNNIsLtTQPYFDSY.L	S6(OGlycan/568.2116); T8(OGlycan/1062.3864)	S,T	707,709	55.20 - 55.20	HexNAc(2)Hex(1),HexNAc(3)Hex(1)NeuAc(1)
320	Y.VLNNIsLtTQPY.F	S6(OGlycan/714.2695); T8(OGlycan/494.1748)	S,T	707,709	169.38 - 169.38	HexNAc(2)Hex(1)Fuc(1),HexNAc(1)NeuAc(1)

321	Y.VLNNIsLtTQPYFDSY.L	S6(OGlycan/730.2644); T8(OGlycan/818.2804)	S,T	707,709	131.68 - 131.68	HexNAc(2)Hex(2),HexNAc(1)Hex(2)NeuAc(1)
322	Y.VLNNIsLtTQPYFDSY.L	S6(OGlycan/656.2276); T8(OGlycan/947.3230)	S,T	707,709	201.79 - 201.79	HexNAc(1)Hex(1)NeuAc(1), HexNAc(1)Hex(1)NeuAc(2)
323	Y.VLNNIsLtTQPY.F	S6(OGlycan/511.1901); T8(OGlycan/656.2276)	S,T	707,709	170.65 - 170.65	HexNAc(1)Hex(1)Fuc(1),HexNAc(1)Hex(1)NeuAc(1)
324	Y.VLNNIsLTTQPYFDSY.L	S6(OGlycan/947.3230)	S	707	131.43 - 205.11	HexNAc(1)Hex(1)NeuAc(2)
325	Y.VLNNIsLtTQPYFDSY.L	S6(OGlycan/494.1748); T8(OGlycan/714.2695)	S,T	707,709	237.29 - 237.29	HexNAc(2)Hex(1)Fuc(1),HexNAc(1)NeuAc(1)
326	Y.VLNNIsLTTQPY.F	S6(OGlycan/859.3070)	S	707	163.57 - 322.32	HexNAc(2)Hex(1)NeuAc(1)
327	Y.VLNNIsLTTQPY.F	S6(OGlycan/1021.3598)	S	707	168.34 - 323.61	HexNAc(2)Hex(2)NeuAc(1)
328	Y.VLNNIsLTTQPy.F	S6(OGlycan/1062.3864); Y12(Phospho/79.9663)	S,Y	707,713	176.39 - 176.39	HexNAc(3)Hex(1)NeuAc(1)
329	Y.VLNNIsLTTQPYFDSY.L	S6(OGlycan/1167.4177)	S	707	189.62 - 189.62	HexNAc(2)Hex(2)Fuc(1)NeuAc(1)
330	Y.VLNNIsLTTQPYFDSY.L	S6(OGlycan/1312.4552)	S	707	298.67 - 298.67	HexNAc(2)Hex(2)NeuAc(2)
331	Y.VLNNIsLTTQPY.F	S6(OGlycan/1312.4552)	S	707	220.62 - 295.05	HexNAc(2)Hex(2)NeuAc(2)
332	Y.VLNNIsLTTQPY.F	S6(OGlycan/365.1322)	S	707	212.91 - 218.21	HexNAc(1)Hex(1)
333	Y.VLNNIsLTTQPY.F	S6(OGlycan/494.1748)	S	707	457.77 - 457.77	HexNAc(1)NeuAc(1)
334	Y.VLNNIsLTTQPY.F	S6(OGlycan/947.3230)	S	707	183.40 - 372.01	HexNAc(1)Hex(1)NeuAc(2)
335	Y.VLNNISLtTQPYFDSY.L	T8(OGlycan/1167.4177)	T	709	159.82 - 159.82	HexNAc(2)Hex(2)Fuc(1)NeuAc(1)
336	Y.VLNNISLtTQPY.F	T8(OGlycan/1312.4552)	T	709	302.49 - 495.45	HexNAc(2)Hex(2)NeuAc(2)
337	Y.VLNNISLtTQPY.F	T8(OGlycan/494.1748)	T	709	328.07 - 345.55	HexNAc(1)NeuAc(1)

338	Y.VLNNISLttQPYFDSY.L	T8(OGlycan/568.2116); T9(OGlycan/1062.3864)	T,T	709,710	144.40 - 144.40	HexNAc(2)Hex(1),HexNAc(3)Hex(1)NeuAc(1)
339	Y.VLNNISLttQPY.F	T8(OGlycan/818.2804); T9(OGlycan/511.1901)	T,T	709,710	409.97 - 435.11	HexNAc(1)Hex(1)Fuc(1),HexNAc(1)Hex(2)NeuAc(1)
340	Y.VLNNISLttQPY.F	T8(OGlycan/1021.3598); T9(OGlycan/730.2644)	T,T	709,710	295.67 - 295.67	HexNAc(2)Hex(2),HexNAc(2)Hex(2)NeuAc(1)
341	Y.VLNNISLtTQPY.F	T8(OGlycan/1021.3598)	T	709	378.87 - 467.09	HexNAc(2)Hex(2)NeuAc(1)
342	Y.VLNNISLtTQPY.F	T8(OGlycan/947.3230)	T	709	597.04 - 657.77	HexNAc(1)Hex(1)NeuAc(2)
343	Y.VLNNISLTtQPY.F	T9(OGlycan/1312.4552)	T	710	400.87 - 400.87	HexNAc(2)Hex(2)NeuAc(2)
344	Y.VLNNISLTtQPYFDSY.L	T9(OGlycan/947.3230)	T	710	242.30 - 242.30	HexNAc(1)Hex(1)NeuAc(2)
345	F.DSYLGCVFNADNLtDy.S	T14(OGlycan/568.2116); Y16(Phospho/79.9663)	T,Y	728,730	126.28 - 126.28	HexNAc(2)Hex(1)
346	F.NADNLTDYsVSSCALR.M	S9(OGlycan/1021.3598)	S	731	144.42 - 144.42	HexNAc(2)Hex(2)NeuAc(1)
347	Y.SVSsCALR.M	S4(OGlycan/1312.4552)	S	734	243.79 - 326.33	HexNAc(2)Hex(2)NeuAc(2)
348	R.mGSGFCVDYNsPSSSSSR.R	M1(Oxidation/15.9949); S11(OGlycan/1312.4552)	M,S	739,749	249.15 - 401.38	HexNAc(2)Hex(2)NeuAc(2)
349	R.mGsGFCVDYNSPSSSSSR.R	M1(Oxidation/15.9949); S3(OGlycan/947.3230)	M,S	739,741	168.47 - 559.35	HexNAc(1)Hex(1)NeuAc(2)
350	R.mGSGFCVDYNsPSSSSSR.R	M1(Oxidation/15.9949); S11(OGlycan/494.1748)	M,S	739,749	510.82 - 597.84	HexNAc(1)NeuAc(1)
351	R.mGSGFCVDYNSPssSSSR.R	M1(Oxidation/15.9949); S13(OGlycan/494.1748); S14(OGlycan/494.1748)	M,S,S	739,751,752	153.75 - 153.75	HexNAc(1)NeuAc(1),HexNAc(1)NeuAc(1)
352	R.mGSGFCVDYNSPSssSSR.R	M1(Oxidation/15.9949); S14(OGlycan/494.1748); S15(OGlycan/494.1748)	M,S,S	739,752,753	144.38 - 165.19	HexNAc(1)NeuAc(1),HexNAc(1)NeuAc(1)
353	R.mGSGFCVDYNSPSSsSSR.R	M1(Oxidation/15.9949); S15(OGlycan/494.1748)	M,S	739,753	637.89 - 637.89	HexNAc(1)NeuAc(1)

354	R.mGSGFCVDYNsPSSSSSR.R	M1(Oxidation/15.9949); S11(OGlycan/656.2276)	M,S	739,749	458.44 - 604.30	HexNAc(1)Hex(1)NeuAc(1)
355	R.mGSGFCVDYNsPSSSSSR.R	M1(Oxidation/15.9949); S11(OGlycan/947.3230)	M,S	739,749	521.13 - 626.88	HexNAc(1)Hex(1)NeuAc(2)
356	R.mGsGFCVDYNSPSSSSSR.R	M1(Oxidation/15.9949); S3(OGlycan/656.2276)	M,S	739,741	462.89 - 462.89	HexNAc(1)Hex(1)NeuAc(1)
357	R.mGsGFCVDY.N	M1(Oxidation/15.9949); S3(OGlycan/947.3230)	M,S	739,741	150.11 - 266.37	HexNAc(1)Hex(1)NeuAc(2)
358	R.MGsGFCVDYNSPSSSSSR.R	S3(OGlycan/947.3230)	S	741	65.81 - 435.39	HexNAc(1)Hex(1)NeuAc(2)
359	R.MGsGFCVDYNSPSSSSSR.R	S3(OGlycan/656.2276)	S	741	126.49 - 264.01	HexNAc(1)Hex(1)NeuAc(1)
360	R.MGsGFCVDYNSPSSSSSR.R	S3(OGlycan/1312.4552)	S	741	76.69 - 497.19	HexNAc(2)Hex(2)NeuAc(2)
361	R.MGsGFCVDYNSPSSSSSR.R	S3(OGlycan/1167.4177)	S	741	357.74 - 357.74	HexNAc(2)Hex(2)Fuc(1)NeuAc(1)
362	R.MGsGFCVDY.N	S3(OGlycan/947.3230)	S	741	121.25 - 169.68	HexNAc(1)Hex(1)NeuAc(2)
363	F.CVDyNsPsSSSSR.R	Y4(Phospho/79.9663); S6(OGlycan/511.1901); S8(OGlycan/494.1748)	Y,S,S	747,749,751	61.03 - 61.03	HexNAc(1)Hex(1)Fuc(1),HexNAc(1)NeuAc(1)
364	F.CVDyNsPSSSSSR.R	Y4(Phospho/79.9663); S6(OGlycan/656.2276)	Y,S	747,749	57.50 - 237.06	HexNAc(1)Hex(1)NeuAc(1)
365	F.CVDyNsPSSSSSR.R	Y4(Phospho/79.9663); S6(OGlycan/859.3070)	Y,S	747,749	79.60 - 79.60	HexNAc(2)Hex(1)NeuAc(1)
366	Y.nsPsSSSSR.R	N1(Deamidated/0.9840); S2(OGlycan/568.2116); S4(OGlycan/947.3230)	N,S,S	748,749,751	149.11 - 194.69	HexNAc(2)Hex(1),HexNAc(1)Hex(1)NeuAc(2)
367	Y.nsPsSSSR.R	N1(Deamidated/0.9840); S2(OGlycan/947.3230); S4(OGlycan/568.2116)	N,S,S	748,749,751	163.75 - 191.23	HexNAc(2)Hex(1),HexNAc(1)Hex(1)NeuAc(2)
368	F.CVDYnSPSSSSsR.R	N5(Deamidated/0.9840); S12(OGlycan/365.1322)	N,S	748,755	162.13 - 162.13	HexNAc(1)Hex(1)
369	F.CVDYnsPSSSSSR.R	N5(Deamidated/0.9840); S6(OGlycan/656.2276)	N,S	748,749	315.87 - 315.87	HexNAc(1)Hex(1)NeuAc(1)

370	F.CVDYnsPSSSSSR.R	N5(Deamidated/0.9840); S6(OGlycan/947.3230)	N,S	748,749	221.15 - 221.15	HexNAc(1)Hex(1)NeuAc(2)
371	F.CVDYnsPSSSSSR.R	N5(Deamidated/0.9840); S6(OGlycan/1167.4177)	N,S	748,749	129.93 - 129.93	HexNAc(2)Hex(2)Fuc(1)NeuAc(1)
372	Y.nsPSSSSSR.R	N1(Deamidated/0.9840); S2(OGlycan/1062.3864)	N,S	748,749	165.04 - 165.04	HexNAc(3)Hex(1)NeuAc(1)
373	R.MGSGFCVDYNsPSSSSSR.R	S11(OGlycan/1312.4552)	S	749	465.91 - 511.72	HexNAc(2)Hex(2)NeuAc(2)
374	Y.NsPssSSSR.R	S2(Phospho/79.9663); S4(OGlycan/859.3070); S5(OGlycan/1062.3864)	S,S,S	749,751,752	145.16 - 145.16	HexNAc(2)Hex(1)NeuAc(1), HexNAc(3)Hex(1)NeuAc(1)
375	F.CVDYNsPSSSSSRR.K	S6(OGlycan/947.3230)	S	749	166.74 - 223.34	HexNAc(1)Hex(1)NeuAc(2)
376	F.CVDYNsPSSSSSR.R	S6(OGlycan/494.1748)	S	749	217.11 - 869.63	HexNAc(1)NeuAc(1)
377	R.MGSGFCVDYNsPSSSSSR.R	S11(OGlycan/494.1748)	S	749	312.36 - 553.97	HexNAc(1)NeuAc(1)
378	F.CVDYNsPSSSSSR.R	S6(OGlycan/1312.4552)	S	749	183.14 - 728.45	HexNAc(2)Hex(2)NeuAc(2)
379	F.CVDYNsPSSSSSR.R	S6(OGlycan/365.1322)	S	749	359.12 - 466.27	HexNAc(1)Hex(1)
380	F.CVDYNsPSSSSSR.R	S6(OGlycan/656.2276)	S	749	379.94 - 701.99	HexNAc(1)Hex(1)NeuAc(1)
381	F.CVDYNsPSSSSSR.R	S6(OGlycan/947.3230)	S	749	187.00 - 757.06	HexNAc(1)Hex(1)NeuAc(2)
382	R.MGSGFCVDYNsPSSSSSR.R	S11(OGlycan/947.3230)	S	749	169.71 - 831.70	HexNAc(1)Hex(1)NeuAc(2)
383	R.MGSGFCVDYNsPSSSSSR.R	S11(OGlycan/656.2276)	S	749	361.37 - 649.77	HexNAc(1)Hex(1)NeuAc(1)
384	Y.NsPsSSSSR.R	S2(OGlycan/494.1748); S4(OGlycan/656.2276)	S,S	749,751	83.39 - 83.39	HexNAc(1)Hex(1)NeuAc(1), HexNAc(1)NeuAc(1)
385	F.CVDYNsPsSSSSR.R	S6(OGlycan/527.1850); S8(Phospho/79.9663)	S,S	749,751	83.30 - 83.30	HexNAc(1)Hex(2)
386	F.CVDYNsPSSSSSR.R	S6(OGlycan/1021.3598)	S	749	205.86 - 499.05	HexNAc(2)Hex(2)NeuAc(1)

387	F.CVDYNsPSSSSSR.R	S6(OGlycan/1167.4177)	S	749	303.24 - 657.44	HexNAc(2)Hex(2)Fuc(1)NeuAc(1)
388	F.CVDYNsPsSSSSR.R	S6(Phospho/79.9663); S8(Phospho/79.9663)	S,S	749,751	293.76 - 293.76	
389	Y.NsPSSSSSR.R	S2(OGlycan/947.3230)	S	749	500.88 - 504.62	HexNAc(1)Hex(1)NeuAc(2)
390	F.CVDYNsPSSSSSR.R	S6(OGlycan/1062.3864)	S	749	204.05 - 204.05	HexNAc(3)Hex(1)NeuAc(1)
391	F.CVDYNsPsSSSSR.R	S6(OGlycan/494.1748); S8(OGlycan/527.1850)	S,S	749,751	208.44 - 208.44	HexNAc(1)Hex(2),HexNAc(1)NeuAc(1)
392	F.CVDYNSPsSSSSR.R	S8(OGlycan/1312.4552)	S	751	304.06 - 536.78	HexNAc(2)Hex(2)NeuAc(2)
393	F.CVDYNSPsSSSSR.R	S8(OGlycan/365.1322)	S	751	400.24 - 422.31	HexNAc(1)Hex(1)
394	F.CVDYNSPsSSSSR.R	S8(OGlycan/494.1748)	S	751	517.78 - 517.78	HexNAc(1)NeuAc(1)
395	F.CVDYNSPssSSSR.R	S8(OGlycan/494.1748); S9(OGlycan/714.2695)	S,S	751,752	121.93 - 433.37	HexNAc(2)Hex(1)Fuc(1),HexNAc(1)NeuAc(1)
396	F.CVDYNSPsSSSSR.R	S8(OGlycan/656.2276)	S	751	501.62 - 832.02	HexNAc(1)Hex(1)NeuAc(1)
397	F.CVDYNSPssSSSR.R	S8(OGlycan/947.3230); S9(OGlycan/947.3230)	S,S	751,752	295.97 - 295.97	HexNAc(1)Hex(1)NeuAc(2), HexNAc(1)Hex(1)NeuAc(2)
398	F.CVDYNSPssSSSRR.K	S8(OGlycan/947.3230); S9(OGlycan/947.3230)	S,S	751,752	40.87 - 63.22	HexNAc(1)Hex(1)NeuAc(2), HexNAc(1)Hex(1)NeuAc(2)
399	F.CVDYNSPsSSSSR.R	S8(OGlycan/947.3230)	S	751	369.78 - 901.28	HexNAc(1)Hex(1)NeuAc(2)
400	R.MGSGFCVDYNSPsSSSSR.R	S13(OGlycan/947.3230)	S	751	545.93 - 545.93	HexNAc(1)Hex(1)NeuAc(2)
401	F.CVDYNSPsSSSSR.R	S8(OGlycan/1062.3864)	S	751	420.46 - 420.46	HexNAc(3)Hex(1)NeuAc(1)
402	F.CVDYNSPsSSSSR.R	S8(OGlycan/1167.4177)	S	751	644.27 - 644.27	HexNAc(2)Hex(2)Fuc(1)NeuAc(1)
403	F.CVDYNSPssSSSR.R	S8(OGlycan/714.2695); S9(OGlycan/494.1748)	S,S	751,752	329.54 - 329.54	HexNAc(2)Hex(1)Fuc(1),HexNAc(1)NeuAc(1)

404	F.CVDYNSPSsSSS R.R	S9(OGlycan/1312.4552)	S	752	603.27 - 668.61	HexNAc(2)Hex(2)NeuAc(2)
405	F.CVDYNSPSsSSS R.R	S9(OGlycan/656.2276)	S	752	636.49 - 636.49	HexNAc(1)Hex(1)NeuAc(1)
406	F.CVDYNSPSsSSS R.R	S9(OGlycan/947.3230)	S	752	858.33 - 882.64	HexNAc(1)Hex(1)NeuAc(2)
407	F.CVDYNSPSSsSS R.R	S10(OGlycan/494.1748)	S	753	810.70 - 810.70	HexNAc(1)NeuAc(1)
408	F.CVDYNSPSSssSR .R	S10(OGlycan/714.2695); S11(OGlycan/730.2644)	S,S	753,754	56.56 - 56.56	HexNAc(2)Hex(2),HexNAc(2)Hex(1)Fuc(1)
409	F.CVDYNSPSSssSR .R	S10(OGlycan/947.3230); S11(OGlycan/656.2276)	S,S	753,754	361.59 - 361.59	HexNAc(1)Hex(1)NeuAc(1), HexNAc(1)Hex(1)NeuAc(2)
410	F.CVDYNSPSSssSR .R	S10(OGlycan/656.2276); S11(OGlycan/947.3230)	S,S	753,754	401.99 - 401.99	HexNAc(1)Hex(1)NeuAc(1), HexNAc(1)Hex(1)NeuAc(2)
411	F.CVDYNSPSSssSR .R	S10(OGlycan/494.1748); S11(OGlycan/714.2695)	S,S	753,754	133.21 - 133.21	HexNAc(2)Hex(1)Fuc(1),He xNAc(1)NeuAc(1)
412	F.CVDYNSPSSSsS R.R	S11(OGlycan/494.1748)	S	754	716.12 - 861.25	HexNAc(1)NeuAc(1)
413	F.CVDYNSPSSSsS R.R	S11(OGlycan/656.2276)	S	754	572.39 - 572.39	HexNAc(1)Hex(1)NeuAc(1)
414	F.CVDYNSPSSSssR .R	S11(OGlycan/947.3230); S12(OGlycan/947.3230)	S,S	754,755	171.26 - 195.02	HexNAc(1)Hex(1)NeuAc(2), HexNAc(1)Hex(1)NeuAc(2)
415	F.CVDYNSPSSSSs R.R	S12(OGlycan/494.1748)	S	755	834.88 - 868.66	HexNAc(1)NeuAc(1)

Table S6. Exposed identified O-glycosylation sites on HKU1 S1

Exposed identified O-glycosylation sites
S36 / S43 (1)
T190 / S194 /T195 (2)
T301
T316
S340
S405 / S406 (1)
T435
S442 / S443 (1)
S492 / S494 (1)
T499
S519 / T526 (1)
S532 / S534 (1)
S562
S578
T665
S731 / S733 / S734 (1)
S741
S749
S751 / S752 / S753 / S754 / S755 (1)
